# Transcriptomic Analysis and Meta-Analysis of Human Granulosa and Cumulus Cells

**DOI:** 10.1371/journal.pone.0136473

**Published:** 2015-08-27

**Authors:** Tanja Burnik Papler, Eda Vrtacnik Bokal, Ales Maver, Andreja Natasa Kopitar, Luca Lovrečić

**Affiliations:** 1 Department of Human Reproduction, Division of Obstetrics and Gynaecology, University Medical Centre Ljubljana, Slajmerjeva 2, Ljubljana, Slovenia; 2 Department of Medical Genetics, Division of Obstetrics and Gynaecology, University Medical Centre, Slajmerjeva 4, Ljubljana, Slovenia; 3 Institute of Microbiology and Immunology, Faculty of Medicine, University of Ljubljana, Zaloska 4, Ljubljana, Slovenia; Clemson University, UNITED STATES

## Abstract

Specific gene expression in oocytes and its surrounding cumulus (CC) and granulosa (GC) cells is needed for successful folliculogenesis and oocyte maturation. The aim of the present study was to compare genome-wide gene expression and biological functions of human GC and CC. Individual GC and CC were derived from 37 women undergoing IVF procedures. Gene expression analysis was performed using microarrays, followed by a meta-analysis. Results were validated using quantitative real-time PCR. There were 6029 differentially expressed genes (q < 10^−4^); of which 650 genes had a log2 FC ≥ 2. After the meta-analysis there were 3156 genes differentially expressed. Among these there were genes that have previously not been reported in human somatic follicular cells, like prokineticin 2 (*PROK2*), higher expressed in GC, and pregnancy up-regulated nonubiquitous CaM kinase (*PNCK*), higher expressed in CC. Pathways like inflammatory response and angiogenesis were enriched in GC, whereas in CC, cell differentiation and multicellular organismal development were among enriched pathways. In conclusion, transcriptomes of GC and CC as well as biological functions, are distinctive for each cell subpopulation. By describing novel genes like *PROK2* and *PNCK*, expressed in GC and CC, we upgraded the existing data on human follicular biology.

## Introduction

Precise and timely coordination between oocyte maturation and the process of somatic cell proliferation and differentiation is crucial for the proper development of the ovarian follicle [1]. Granulosa cells (GC) differentiate into two functionally distinct groups during ovarian follicle growth: cumulus cells (CC) that are in physical contact with the oocyte, and mural GC, which line the wall of the antral follicle [2]. Bidirectional communication between the oocyte and its surrounding somatic cells through paracrine signalling and gap junctions is required during folliculogenesis for the development of follicular compartments, oocyte maturation and competence acquisition [3].

In order to secure its own optimal growth and maturation [4], the oocyte regulates the differentiation, proliferation, apoptosis and luteinisation of GC and CC by secretion of paracrine factors such as growth differentiation factor 9 (*GDF-9*) and bone morphogenetic protein 15 (*BMP-15*) [5]. *GDF-9* and *BMP-15* activate signalling pathways and induce GC gene expression which leads to a functional change of these cells necessary for oocyte development [6].

On the other hand, somatic follicular cells provide support for oocyte growth, resumption of meiosis, nuclear and cytoplasmic maturation and control oocyte's transcriptional activity through the activation of different signalling pathways [7]. For example, in mice, GC derived Kit ligand (*KitL*) has been shown to stimulate oocyte growth via activation of phosphatidylinositol 3-kinase (PI3K) pathway [8]. Furthermore, binding of *KitL* to its receptor *Kit* on the oocyte activates Ras, Raf, mitogen activated protein kinase (MAPK) and protein kinase B (PKB/Akt)—apoptosis related signalling pathways [9].

Identification of key molecules and signalling pathways within GC and CC is necessary to gain insight of how the oocyte acquires developmental competence during its growth within the follicle and for uncovering molecular regulators of this process. This could help optimize *in vitro* techniques for oocyte maturation. There have been some studies conducted on human GC and CC in order to provide information on cell-type specific transcriptome [10, 11, 12]. These studies identified specific enriched biological processes in GC (cell cycle, immune response, cell proliferation, steroidogenesis) and CC (cell signalling, protein degradation, angiogenesis). However, our knowledge of the key genes and pathways of human folliculogenesis and oocyte competence acquisition is still incomplete. Identification of specific genes and functions of human GC and CC would help in understanding the functional specifics of each follicular compartment and its role in the process of human folliculogenesis.

The aim of the present study was to identify differentially expressed genes and analyse biological processes in human GC and CC obtained during IVF procedures.

## Materials and Methods

### Patient population and IVF procedure

Thirty-seven (37) IVF patients were included in this study. All patients were less than 35 years old, had a body mass index (BMI) between 17 and 26 kg/m^2^, were enrolled in the IVF procedure due to tubal cause of infertility and had their first or second IVF procedure. Their partner’s spermiogram was normal according to the WHO criteria [13]. The study was approved by the National Medical Ethics Committee of the Republic of Slovenia and written informed consent was obtained from all women prior to participation.

All patients were treated with recombinant FSH (Puregon; Schering Plough, New Jersey, USA) and GnRH antagonist cetrorelix acetate (Cetrotide; Asta Medica AG, Frankfurt, Germany). Vaginal ultrasound examination was performed to monitor follicular development. Final follicular maturation was induced by administering 10,000 IU of human chorionic gonadotropin (hCG) (Pregnyl; N.V. Organon, Oss, the Netherlands) when at least three follicles were ≥17 mm. Ultrasound-guided transvaginal puncture was performed 34–36 h later. Each follicle was aspirated separately. After aspiration, cumulus-oocyte complexes (COC) were removed from the follicular fluid. Cumulus cells of each oocyte were removed immediately. Classical IVF was used for oocyte fertilization. Embryo transfer was performed on day 5 after oocyte retrieval. The GC and CC samples in this study were all derived from follicles containing mature, MII, oocytes.

### Study design

We first performed genome-wide gene expression analysis using microarrays on 34 individual GC and 30 individual CC samples derived from 21 women. The next step included quantitative real-time PCR (qPCR) validation performed on 19 individual GC and 19 individual CC samples derived from 16 women that were newly included in the study. Data from two previous studies, where comparison of human GC and CC gene expression profiles was performed, were used for the meta-analysis for the identification of consistent differentially expressed genes.

### Samples collection and preparation

Immediately after oocyte retrieval, CC of each oocyte were mechanically removed by a needle and a glass denudation pipette (Swemed, Göteborg, Sweden), washed in phosphate-buffered saline (PBS), snap frozen in liquid nitrogen and stored at -80°C until RNA isolation.

For GC purification, Dynabeads CD45 Magnetic Beads (Invitrogen Dynal AS, Oslo, Norway) were used for the depletion of CD45+ cells according to the manufacturer's protocol with some adjustments. The beads were used to separate leukocyte CD45+ cells from GC to exclude the risk of contamination [14]. Follicular fluids were centrifuged at 800x g at 4°C for 10 minutes. Follicular fluids were then snap frozen in liquid nitrogen and stored at -80°C. The sediment was resuspended in 2 ml of lysis solution (0.15 M NH4Cl) to remove red blood cells, incubated for 10 minutes and centrifuged at 600x g at 4°C for 10 minutes. The supernatant was discarded and the sediment resuspended in 1 ml PBS with 0.1% bovine serum albumin (BSA) and 0.002 M ethylenediaminetetraacetic acid (EDTA). The suspension was added to the tubes containing 50 μl of Dynabeads CD45 and incubated at 4°C for 30 minutes with gentle tilting and rotation of the mixer. The tubes were pre-coated with 2 ml of Roswell Park Memorial Institute medium (RPMI) with 1% fetal calf serum (FCS) for 5 minutes. After incubation, the samples were diluted with PBS with 0.1% BSA and 0.002 M EDTA to 4 ml and placed in a magnet for 10 minutes. The supernatant was transferred to a new pre-coated tube and centrifuged at 800x g at 4°C for 15 minutes. The supernatant was discarded and sediment resuspended in 1 ml of PBS. The samples were then centrifuged at 2000 x g for 5 minutes, supernatant discharged and sediment snap frozen in liquid nitrogen and stored at -80°C. The purity of the samples was tested with flow cytometry. The samples were prepared from a GC suspension before isolation and compared to cell suspension after CD45+ depletion. The contamination with leucocytes was investigated using antibodies CD45 conjugated with Fluorescin Isothiocyanate (FITC) and CD14 Phycoerythrin (PE) (both from BD Bioscience, San Jose, USA). Samples were washed and resuspended in PBS with 1% BSA and incubated with antibodies for 20 minutes. The purity of the samples was characterized by Forward Scatter (FSC) versus Side Scatter (SSC) followed by gating on CD45 and CD14. Data were collected on FACSCanto flow cytometer (BD Bioscience, San Jose, USA) and expression of various markers was assessed using FlowJo analysis software (TreeStarInc, Ashland, USA).

Before depletion of CD45+ cells there were, in average, 14.8% of granulocytes, 9.7% of lymphocytes, 9.7% of monocytes and 66.9% of GC present. After the depletion, the proportions of leukocytes were significantly lower. In average, there were 4.7% of granulocytes, 1.8% of monocytes, 1.7% of lymphocytes and 91.8% of GC. Thus, we have achieved a sufficient purity of GC samples, as it has been established that a gene expression profile of a more than 75% pure sample was indistinguishable from a 100% pure sample [15].

### RNA extraction and cDNA synthesis

TRI reagent (Sigma—Aldrich, St.Louis, USA) was used for RNA extraction according to a slightly modified manufacturer’s protocol. Due to a small sample volume, glycogen was used as a carrier to increase RNA yield. Individual GC and CC samples were homogenized in 500 μL TRI reagent supplemented with 125 μg of glycogen (Ambion, Austin, USA). After 2 min incubation at room temperature, 100 μL of chloroform was added and the sample was vortexed vigorously. RNA was precipitated from the aqueous phase with isopropanol and collected after 15 min of centrifugation at 12,000x g and 4°C. RNA pellet was washed three times with 75% ethanol, dried and dissolved in 15 μL of RNAse free water. The integrity of the RNA samples was assessed with an Agilent 2100 Bioanalyzer (Agilent, Palo Alto, USA) and the total RNA quantity was measured with a Nanodrop ND-1000 spectrophotometer (Nanodrop Technologies, Wilmington, USA). SuperScript Vilo reverse transcriptase (Invitrogen, Carlsbad, USA) was used for cDNA synthesis from 150 ng RNA according to the manufacturer’s instructions.

### Microarray analysis

Microarray profiling of global gene expression on RNA samples from GC and CC was performed using Agilent SurePrint G3 Human gene expression 8x60K two-color microarrays (Agilent eArray design identifier: 028004), with 42.405 unique probes targeting for most of the RefSeq mRNA sequences. The experimental design comprised of co-hybridization of each tested sample with common universal reference RNA sample (Universal Human Reference RNA from Agilent). The labeling procedure was performed using Agilent’s two-color Low Input Quick Amp Labeling Kit (two-color) according to manufacturers’ instructions, labeling test samples with Cy3 and reference samples with Cy5 dye.

Microarray slides were scanned using Agilent High Resolution Microarray Scanner System after hybridization, using the manufacturer’s recommended scanning settings. Subsequently, microarray features were extracted using Agilent Feature Extraction (FE) software v10.7.3.1. Background was subtracted using background de-trending algorithm implemented in FE software, features with non-uniform fluorescence profiles were removed from further analyses and linear lowess intra-array normalization was performed to correct for the presence of dye bias. The consistency of log ratio values was inspected using manufacturers provided spike-in probes and reproducibility was evaluated by investigating the extent of variance in the population of replicated probes on the array. The microarray data are available at the Gene Expression Omnibus (GEO) public repository (http://www.ncbi.nlm.nih.gov/geo) under the accession number GSE55654.

### Statistical analysis of microarray data

All the steps described in this section, including post-processing, statistical comparisons and classification performance estimations were performed using packages from Bioconductor v2.8 project in R statistical environment version 2.13.1.

Processed fluorescence values obtained by feature extraction were imported and inspected for presence of missing values and other irregularities. Probe annotations were obtained from the Agilent’s eArray service (*earray*.*chem*.*agilent*.*com*). Prior to the calculation of Cy3/Cy5 log2 ratios, fluorescence values were offset by 100 units to prevent an undesirable increase in log2 ratio variance in the population of features with low fluorescence values. Quality assessment of microarray hybridizations was performed by inspecting the signal distribution in box and MA plots. Principal component analysis was done to identify the presence of possible batch and other confounding effects.

Statistical comparisons of expression values were evaluated using moderated t-test approach implemented in limma [16]. Significance values and log2 fold-change were calculated and afterwards, *p*-values were controlled for multiple testing effect by the method described by Benjamini and Hochberg [17] and implemented into the limma workflow.

### Meta-analysis

Two datasets that analysed global gene expression profiles in human GC and CC samples with accession numbers GSE18559 [10] and E-MEXP-3641 [11], together with our own data. were included in the meta-analysis. Raw data from microarray experiments were obtained from Gene Expression Omnibus (GEO) repository (http://www.ncbi.nlm.nih.gov/geo/) [18] and from ArrayExpress repository (www.ebi.ac.uk/arrayexpress/).

Raw datasets were put through quality control steps implemented in arrayQualityMetrics package, followed by intra-array quantile and inter-array loess normalization, where necessary. Afterwards, intensities of probe replicates targeting specific transcripts were median averaged and only values for transcripts interrogated in all three studies were retained through further meta-analysis steps.

Differential expression of genes across all three studies was calculated using meta-analysis algorithms implemented in the RankProd package [19]. RankProd uses a non-parametric approach that selects genes displaying consistently highly ranked across different microarray studies and is therefore a feasible meta-analytic tool across a variety of microarray studies originating from different laboratories and performed under differing conditions [19]. We utilized RPadvance function, with origin parameter set to account for data originating from three different sources. P values were calculated using permutation approach (1000 permutations) and predicted false positive rates estimated across permutation cycles [20].

### Gene ontology and IPA analysis

Gene ontology (GO) analysis was performed to define enriched, cell specific GO profiles using Gene Codis [21]. We uploaded our two sets of genes: one set of 524 genes that were up-regulated in GC and one set of 126 that were up-regulated in CC. All of the selected genes had a log_2_ fold difference > ± 2 and *q*-value < 10^−4^ so they were considered significantly differentially expressed. Up-regulation of genes in GC population, as mentioned in this manuscript, refers to down-regulation of genes in CC and vice versa.

Our list of significantly differentially expressed genes (log_2_ FC > ±2, *q*-value < 10^−4^) was subjected to Ingenuity Pathway Analysis (IPA) Software (Ingenuity Systems Inc, Redwood City, USA) for network enrichment analysis in each cell subpopulation.

### Quantitative real-time PCR analysis

We performed validation of microarray data results using qPCR on an independent set of GC and CC samples. The genes selected for validation were angiotensin I converting enzyme 2 (*ACE2* **)**, dual specificity phosphatase 6 (*DUSP6*), fibrinogen gamma chain (*FGG*), HtrA serine peptidase 1 (*HTRA1*), interleukin 1, beta (*IL1B*), pregnancy up-regulated nonubiquitous CaM kinase (*PNCK*), prokineticin 2 (*PROK2*), ryanodine receptor 2 (cardiac) (*RYR2*).

Pre-designed TaqMan Gene Expression assays (Applied Biosystems, Foster City, CA, USA) were used for mRNA quantification. All reactions were performed in triplicates in 96-well formats in a 20 μl final volume. The measurements were performed on ABI Prism 7000 Sequence detection system (Applied Biosystems, Foster City, USA). Thermal cycling conditions were as follows: an initial step at 50°C for 2 min, denaturation at 95°C for 10 min, amplification for 40 cycles at 95°C for 15s and at 60°C for 1 min. The threshold cycle (Ct) values were then determined for each assay and normalized to internal control glyceraldehyde-3-phosphate dehydrogenase (*GAPDH*) that was co-ran with each sample. Differences in gene expression were calculated using the delta-delta Ct method, as previously described by Livak and Schmittgen [22]. The significance of the mean relative gene expression level differences was calculated using two-sample t-distribution test, differences were considered significant at α < 0.05.

## Results

### Global differential gene expression

**Fig 1:**
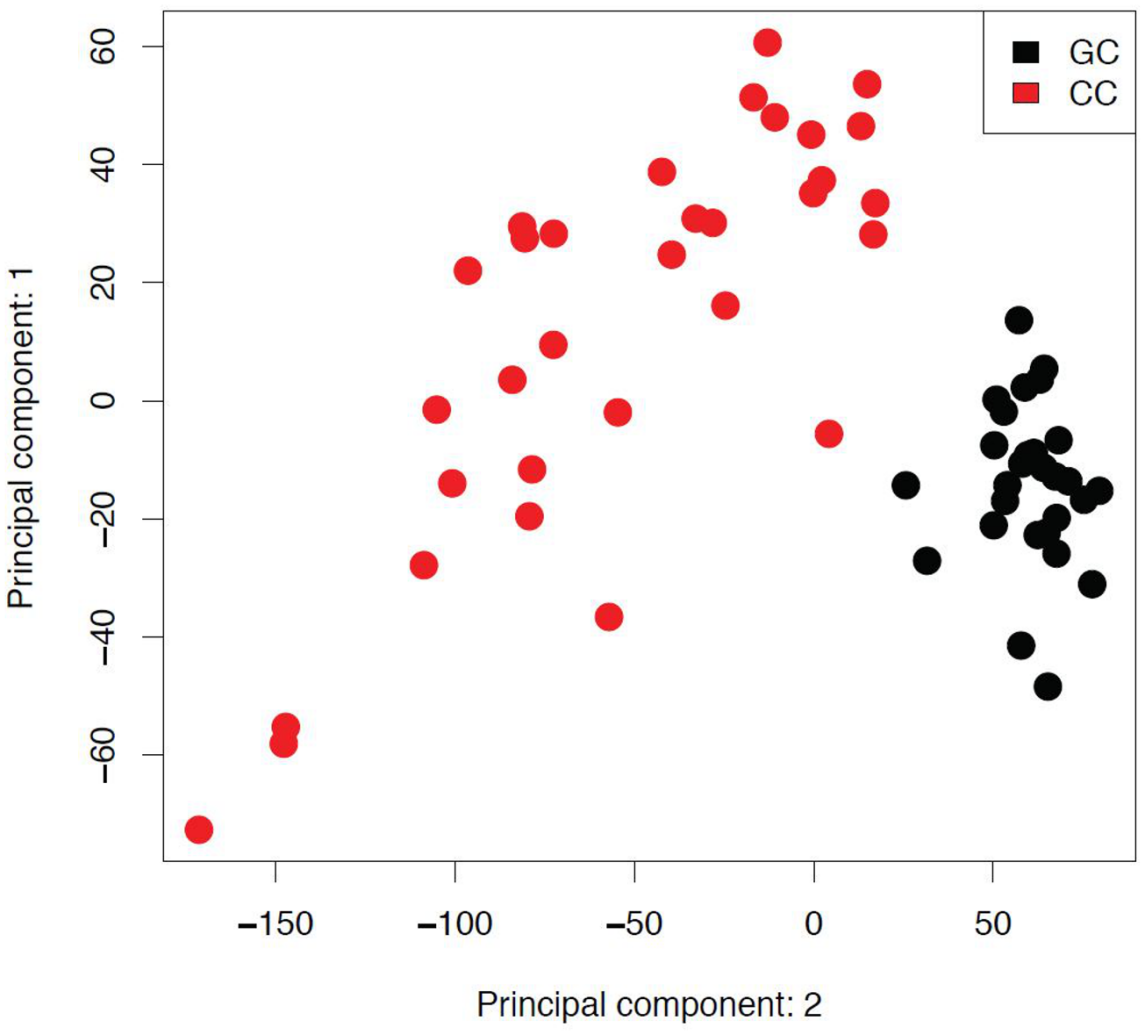
Principal component analysis between granulosa and cumulus cells. The axes of the plot represent first two main components of variance, obtained by performing principal component analysis (PCA) on global expression profiles. GC- granulosa cells; CC- cumulus cells.

The comparison of global gene expression profiles of GC and CC showed cell type specific profiles. Complete transcriptional profiles of the two cell populations were subjected to principal component analysis (PCA); first two principal variance components clearly separated expression profiles of GC and CC populations (Fig 1).

There were 6029 genes significantly (*q* < 10^−4^) differentially expressed between GC and CC in our study, with 650 having a log_2_ fold change ≥ 2, with 524 genes higher expressed in GC and 126 genes higher expressed in CC. The lists of top 100 differentially expressed genes are represented in Tables 1 and 2 for GC and CC, respectively. The full list of differentially expressed genes is represented in S1 Table.

**Table 1:** The list of top 100 genes up-regulated in GC.

Gene Symbol	Gene name	Absolute array intensity (log2A)	logFC(CC/GC)	q value
ADAM8	ADAM metallopeptidase domain 8	11.4	-3.30	3.99E-15
AIF1	allograft inflammatory factor 1	12.3	-3.44	2.28E-13
ALAS2	aminolevulinate, delta-, synthase 2	12.7	-3.56	1.09E-12
APBB1IP	amyloid beta	10.4	-3.56	1.78E-15
APOBEC3A	apolipoprotein B mRNA editing enzyme, catalytic polypeptide-like 3A	7.3	-3.29	3.00E-12
APOBR	apolipoprotein B receptor	7.9	-3.30	1.71E-16
AQP9	aquaporin 9	8.3	-3.61	7.09E-15
ARHGAP30	Rho GTPase activating protein 30	7.7	-3.59	1.78E-16
ARHGDIB	Rho GDP dissociation inhibitor	13.5	-3.58	2.76E-14
BCL2A1	BCL2-related protein A1	9.6	-3.52	2.57E-13
C16orf54	chromosome 16 open reading frame 54	8.7	-4.03	4.33E-16
C5AR1	complement component 5a receptor 1	8.1	-4.03	1.27E-17
CAMP	cathelicidin antimicrobial peptide	7.4	-3.26	3.62E-13
CASP1	caspase 1, apoptosis-related cysteine peptidase (interleukin 1, beta, convertase)	9.4	-3.47	2.04E-15
CCL3	chemokine (C-C motif) ligand 3	10.3	-3.22	4.68E-11
CCL5	chemokine (C-C motif) ligand 5	8.3	-3.47	1.65E-15
CCR1	chemokine (C-C motif) receptor 1	9.7	-3.45	2.21E-12
CCR7	chemokine (C-C motif) receptor 7	8.2	-3.47	9.49E-18
CD247	CD247 molecule	6.4	-3.28	3.01E-17
CD300A	CD300a molecule	7.9	-3.20	8.23E-15
CD52	CD52 molecule	11.4	-3.31	8.27E-15
CD53	CD53 molecule	10.2	-3.25	1.48E-14
CD97	CD97 molecule	12.1	-3.48	7.57E-17
CMTM2	CKLF-like MARVEL transmembrane domain containing 2	7.6	-3.50	8.96E-15
CX3CR1	chemokine (C-X3-C motif) receptor 1	9.9	-3.49	4.10E-15
CYTH4	cytohesin 4	6.4	-3.28	1.40E-16
CYTIP	cytohesin 1 interacting protein	9.4	-3.39	5.52E-14
DEFA3	defensin, alpha 3, neutrophil-specific	8.5	-4.01	2.00E-13
ENST00000390547	immunoglobulin heavy constant alpha 1 [Source:HGNC Symbol;Acc:5478] [ENST00000390547]	9.1	-3.23	2.60E-12
EVI2B	ecotropic viral integration site 2B	8.8	-3.72	3.31E-14
FAM65B	family with sequence similarity 65, member B	7.3	-3.36	1.17E-11
FCAR	Fc fragment of IgA	6.9	-3.36	1.09E-16
FCGR2A	Fc fragment of IgG, low affinity IIa, receptor	10.0	-3.37	5.60E-15
FCGR2A	Fc fragment of IgG, low affinity IIa, receptor (CD32)	10.0	-3.50	4.91E-14
FGD3	FYVE, RhoGEF and PH domain containing 3	9.5	-3.38	4.21E-18
FOSB	FBJ murine osteosarcoma viral oncogene homolog B	9.8	-3.65	1.27E-18
FPR2	formyl peptide receptor 2	7.4	-3.40	1.68E-13
GABRP	gamma-aminobutyric acid	8.8	-3.85	6.37E-15
GIMAP4	GTPase, IMAP family member 4	7.5	-3.27	2.32E-13
GLT1D1	glycosyltransferase 1 domain containing 1	8.7	-3.31	9.42E-14
GMFG	glia maturation factor, gamma	12.6	-3.72	4.86E-15
GZMA	granzyme A (granzyme 1, cytotoxic T-lymphocyte-associated serine esterase 3)	7.7	-3.70	5.46E-16
GZMB	granzyme B (granzyme 2, cytotoxic T-lymphocyte-associated serine esterase 1)	8.6	-3.45	8.26E-18
HBA2	hemoglobin, alpha 2	16.5	-4.89	2.77E-14
HBM	hemoglobin, mu	9.5	-3.56	4.72E-16
HBQ1	hemoglobin, theta 1	11.7	-4.11	1.87E-15
HCAR3	hydroxycarboxylic acid receptor 3	9.1	-3.42	6.30E-14
HCK	hemopoietic cell kinase	7.7	-3.26	6.18E-16
HCLS1	hematopoietic cell-specific Lyn substrate 1	12.3	-3.73	1.16E-16
IL18RAP	interleukin 18 receptor accessory protein	7.7	-3.42	1.55E-13
IL1B	interleukin 1, beta	9.3	-3.67	4.64E-15
IL2RB	interleukin 2 receptor, beta	7.8	-3.31	2.40E-18
INPP5D	inositol polyphosphate-5-phosphatase	10.5	-3.66	3.37E-17
ITGAL	integrin, alpha L (antigen CD11A (p180), lymphocyte function-associated antigen 1; alpha polypeptide)	8.3	-3.27	8.87E-17
ITGB2	integrin, beta 2 (complement component 3 receptor 3 and 4 subunit)	7.1	-3.67	6.33E-16
KLF2	Kruppel-like factor 2 (lung)	10.6	-4.05	1.54E-18
KLRB1	killer cell lectin-like receptor subfamily B, member 1	7.3	-3.42	1.04E-14
KRT1	keratin 1	8.2	-3.42	9.50E-09
LAPTM5	lysosomal protein transmembrane 5	10.0	-3.39	1.80E-13
LCP1	lymphocyte cytosolic protein 1 (L-plastin)	12.6	-3.43	1.71E-13
lincRNA:chr1:205404902–205417627_F	lincRNA:chr1:205404902–205417627 forward strand	7.5	-3.38	6.18E-14
lincRNA:chr10:17250419–17261819_F	lincRNA:chr10:17250419–17261819 forward strand	8.7	-3.55	2.86E-15
lincRNA:chr15:52209683–52223508_R	lincRNA:chr15:52209683–52223508 reverse strand	7.9	-3.37	1.42E-10
lincRNA:chr17:29887612–29925137_F	lincRNA:chr17:29887612–29925137 forward strand	7.7	-3.72	1.80E-13
lincRNA:chr17:73598283–73599500_F	lincRNA:chr17:73598283–73599500 forward strand	10.4	-3.73	1.17E-12
lincRNA:chr4:769425–775573_F	lincRNA:chr4:769425–775573 forward strand	11.6	-3.76	8.00E-14
lincRNA:chr5:12625075–12747025_F	lincRNA:chr5:12625075–12747025 forward strand	11.2	-3.98	4.90E-15
LOC100128348	cDNA FLJ46249 fis, clone TESTI4021377	6.1	-3.25	1.26E-18
LOC100133286	uncharacterized LOC100133286	9.2	-3.29	6.98E-13
LOC100652730	Homo sapiens hypothetical LOC100652730 (LOC100652730), miscRNA	7.0	-3.21	3.98E-12
LSP1	lymphocyte-specific protein 1	10.4	-3.42	1.82E-17
LTB	lymphotoxin beta (TNF superfamily, member 3)	9.1	-4.01	6.09E-18
LYN	v-yes-1 Yamaguchi sarcoma viral related oncogene homolog	10.6	-3.59	9.89E-15
MBP	myelin basic protein	6.3	-3.40	1.06E-13
MNDA	myeloid cell nuclear differentiation antigen	8.1	-3.35	1.62E-11
MPEG1	macrophage expressed 1	7.6	-3.21	8.07E-15
NCF1	neutrophil cytosolic factor 1	8.5	-3.21	1.89E-17
NCF2	neutrophil cytosolic factor 2	8.7	-3.44	2.04E-15
NFAM1	NFAT activating protein with ITAM motif 1	8.4	-3.44	1.54E-16
NFE2	nuclear factor (erythroid-derived 2)	11.8	-3.55	1.07E-13
PLEK	Pleckstrin	7.8	-3.40	2.19E-15
PREX1	phosphatidylinositol-3,4,5-trisphosphate-dependent Rac exchange factor 1	11.2	-3.49	3.03E-15
PROK2	prokineticin 2	8.2	-4.29	5.90E-14
PTPRC	protein tyrosine phosphatase, receptor type, C	10.5	-3.38	3.77E-11
PTPRC	tyrosine phosphatase, receptor type, C	10.5	-3.63	4.10E-16
RASSF5	Ras association (RalGDS/AF-6) domain family member 5	6.9	-3.24	3.81E-15
RGL4	ral guanine nucleotide dissociation stimulator-like 4	8.0	-3.42	5.30E-15
S100A12	S100 calcium binding protein A12	9.3	-3.68	6.60E-14
S100A4	S100 calcium binding protein A4	13.1	-3.32	1.44E-14
S1PR4	sphingosine-1-phosphate receptor 4	9.8	-3.77	1.61E-16
SECTM1	secreted and transmembrane 1	10.8	-3.59	3.72E-16
SORL1	sortilin-related receptor, L(DLR class) A repeats containing	12.4	-3.47	8.10E-13
SPI1	spleen focus forming virus (SFFV) proviral integration oncogene spi1	9.3	-3.22	2.42E-19
TAGAP	T-cell activation RhoGTPase activating protein	7.6	-3.81	6.17E-17
TBC1D10C	TBC1 domain family, member 10C	10.3	-3.39	8.67E-16
TLR1	toll-like receptor 1	8.3	-3.36	2.16E-12
TNFSF10	tumor necrosis factor (ligand) superfamily, member 10	8.6	-3.43	1.18E-11
TRIM58	tripartite motif containing 58	9.6	-3.36	4.32E-11
TTTY16	testis-specific transcript, Y-linked 16 (non-protein coding)	9.0	-4.03	1.06E-14
VWA1	von Willebrand factor A domain containing 1	11.1	-3.45	6.99E-14

**Table 2:** The list of top 100 genes up-regulated in CC.

Gene Symbol	Gene name	Absolute array intensity (log2A)	logFC(CC/GC)	q value
ACE2	angiotensin I converting enzyme (peptidyl-dipeptidase A) 2	8.7	3.39	3.04E-20
ACOXL	acyl-CoA oxidase-like	8.3	3.12	2.36E-23
ACTA1	actin, alpha 1, skeletal muscle	7.9	2.71	5.34E-14
ADAMTS4	ADAM metallopeptidase with thrombospondin type 1 motif, 4	6.1	2.57	2.17E-18
ALPK2	alpha-kinase 2	7.7	2.37	2.26E-18
AMH	anti-Mullerian hormone	8.8	3.15	1.80E-19
BEX1	brain expressed, X-linked 1	13.2	3.23	8.97E-23
BMPER	BMP binding endothelial regulator	6.0	3.18	3.15E-26
BUB1	budding uninhibited by benzimidazoles 1 homolog	9.7	2.26	2.51E-19
C1orf141	chromosome 1 open reading frame 141	7.4	2.44	2.13E-16
C2CD4B	C2 calcium-dependent domain containing 4B	7.1	2.64	7.96E-18
CACNA1C	calcium channel, voltage-dependent, L type, alpha 1C subunit	9.9	2.33	2.31E-16
CBLN1	cerebellin 1 precursor	6.9	2.51	4.18E-13
CDH11	cadherin 11, type 2, OB-cadherin	9.7	2.24	1.76E-13
CHGA	chromogranin A (parathyroid secretory protein 1)	7.4	2.57	3.04E-24
CHGB	chromogranin B (secretogranin 1)	8.5	2.23	8.80E-14
CIB4	calcium and integrin binding family member 4	7.2	2.87	3.69E-19
CILP	cartilage intermediate layer protein, nucleotide pyrophosphohydrolase	8.4	3.09	1.33E-17
CLSTN2	calsyntenin 2	8.3	2.26	2.26E-12
CORO2A	coronin, actin binding protein, 2A	9.3	2.60	1.25E-22
CTSK	cathepsin K	10.9	2.77	1.97E-16
CYP19A1	cytochrome P450, family 19, subfamily A, polypeptide 1	7.2	2.32	4.76E-13
CYP19A1	cytochrome P450, family 19, subfamily A, polypeptide 1	7.2	2.26	7.06E-18
DAPL1	death associated protein-like 1	7.0	3.51	1.69E-12
DAPL1	death associated protein-like 1	7.0	2.72	1.35E-12
DHH	desert hedgehog	7.7	2.39	2.44E-18
DKFZp451A211	ref|PREDICTED: Homo sapiens DKFZp451A211 protein (DKFZp451A211), mRNA [XM_003403663]	7.7	2.46	3.22E-19
DOK5	docking protein 5	8.7	2.65	4.18E-18
DPYSL4	dihydropyrimidinase-like 4	9.7	2.34	1.65E-12
DUOXA2	dual oxidase maturation factor 2	6.0	2.60	1.32E-13
E2F7	E2F transcription factor 7	11.1	3.43	1.07E-16
E2F7	E2F transcription factor 7	11.1	2.25	3.17E-07
EPHB1	EPH receptor B1	7.1	2.40	1.97E-16
F3	coagulation factor III (thromboplastin, tissue factor)	9.8	2.33	5.27E-15
FABP3	fatty acid binding protein 3, muscle and heart (mammary-derived growth inhibitor)	7.9	2.31	8.86E-18
FAM110C	family with sequence similarity 110, member C	7.3	2.46	3.33E-13
FAM150B	family with sequence similarity 150, member B	6.9	2.92	9.90E-18
FAM150B	family with sequence similarity 150, member B	6.9	2.75	6.21E-17
FAM189A1	family with sequence similarity 189, member A1	8.4	2.26	3.52E-14
FGF11	fibroblast growth factor 11	7.8	2.42	1.26E-18
FN1	fibronectin 1	10.4	2.51	2.14E-14
FOXG1	forkhead box G1	8.2	3.30	5.27E-14
GABBR2	gamma-aminobutyric acid (GABA) B receptor, 2	7.8	2.46	2.75E-12
GAL	galanin prepropeptide	12.2	2.92	9.22E-15
GAP43	growth associated protein 43	7.4	2.36	7.87E-22
GDF6	growth differentiation factor 6	8.7	3.36	1.80E-19
GJA5	gap junction protein, alpha 5	6.8	2.35	1.24E-16
GRIK3	glutamate receptor, ionotropic, kainate 3	7.2	2.76	6.85E-13
HTRA1	HtrA serine peptidase 1	14.0	3.79	2.99E-20
IGFBP5	insulin-like growth factor binding protein 5	11.3	3.53	3.37E-23
ISM1	isthmin 1 homolog	6.9	2.56	7.96E-18
KCNK3	potassium channel, subfamily K, member 3	8.0	2.66	5.64E-20
KCNT1	potassium channel, subfamily T, member 1	7.2	2.25	1.41E-15
KRTAP13-1	keratin associated protein 13–1	7.1	2.84	9.16E-26
KRTAP13-2	keratin associated protein 13–2	7.5	2.57	1.01E-20
LEFTY2	eft-right determination factor 2	9.3	2.41	2.43E-13
LIMS2	LIM and senescent cell antigen-like domains 2	6.9	2.54	2.88E-20
LOC100506189	ref|PREDICTED: Homo sapiens hypothetical LOC100506189 (LOC100506189), miscRNA [XR_108925]	7.3	2.43	1.14E-15
LOC100506189	ref|PREDICTED: Homo sapiens hypothetical LOC100506189 (LOC100506189), miscRNA [XR_108925]	7.3	2.41	4.79E-12
LOC100506189	ref|PREDICTED: Homo sapiens hypothetical LOC100506189 (LOC100506189), miscRNA [XR_108925]	7.3	2.39	4.79E-15
LRAT	lecithin retinol acyltransferase (phosphatidylcholine—retinol O-acyltransferase)	7.9	2.78	5.40E-15
LTBP1	latent transforming growth factor beta binding protein 1	9.5	2.25	1.19E-16
MT1DP	metallothionein 1D, pseudogene (MT1DP), transcript variant 1	6.9	2.66	4.77E-17
NOS2	nitric oxide synthase 2, inducible	7.3	2.64	2.22E-15
NUDT10	nudix (nucleoside diphosphate linked moiety X)-type motif 10	8.6	2.35	3.58E-13
PCK1	phosphoenolpyruvate carboxykinase 1	8.6	3.22	1.69E-16
PCYT1B	phosphate cytidylyltransferase 1, choline, beta	7.5	2.53	1.24E-17
PDLIM3	PDZ and LIM domain 3	6.2	2.51	1.80E-19
PDZD2	PDZ domain containing 2	8.7	2.22	9.38E-15
PLCXD3	phosphatidylinositol-specific phospholipase C, X domain containing 3	7.5	2.32	2.71E-14
PLOD2	procollagen-lysine, 2-oxoglutarate 5-dioxygenase 2	11.8	2.50	1.01E-18
PNCK	pregnancy up-regulated non-ubiquitously expressed CaM kinase	8.0	3.35	3.88E-22
PPP1R14C	protein phosphatase 1, regulatory (inhibitor) subunit 14C	7.8	2.45	2.14E-15
PRB1	proline-rich protein BstNI subfamily 1	7.9	2.23	1.24E-12
PRB2	proline-rich protein BstNI subfamily 2	8.3	3.44	3.12E-14
PRB4	proline-rich protein BstNI subfamily 4	10.2	2.24	4.59E-12
RHOBTB3	Rho-related BTB domain containing 3	11.3	2.78	9.13E-18
RTN4RL1	reticulon 4 receptor-like 1	8.8	2.37	3.85E-20
RYR2	ryanodine receptor 2	7.6	2.85	6.30E-25
SCG2	secretogranin II	8.4	3.28	9.42E-22
SEMA5B	sema domain, seven thrombospondin repeats (type 1 and type 1-like), transmembrane domain (TM) and short cytoplasmic domain, (semaphorin) 5B	6.9	2.34	7.90E-11
SERPINA3	serpin peptidase inhibitor, clade A (alpha-1 antiproteinase, antitrypsin), member 3	11.4	2.27	1.36E-15
SERPINA5	serpin peptidase inhibitor, clade A (alpha-1 antiproteinase, antitrypsin), member 5	9.4	2.46	1.66E-17
SLC15A1	solute carrier family 15 (oligopeptide transporter), member 1	7.1	2.91	3.11E-18
SLC28A3	solute carrier family 28 (sodium-coupled nucleoside transporter), member 3	7.1	2.50	2.39E-17
SMOC2	SPARC related modular calcium binding 2	10.3	2.73	4.09E-19
SMOC2	SPARC related modular calcium binding 2	10.3	2.45	6.25E-20
SPON2	spondin 2, extracellular matrix protein	13.5	2.42	4.77E-15
ST6GAL2	ST6 beta-galactosamide alpha-2,6-sialyltranferase 2	6.5	3.24	3.87E-24
SYNDIG1	synapse differentiation inducing 1	8.7	3.75	5.40E-25
TAC3	tachykinin 3	9.5	2.80	1.48E-22
TMEM114	transmembrane protein 114	7.1	3.00	4.84E-13
TMEM132C	transmembrane protein 132C	8.0	3.09	5.64E-20
TNC	tenascin C	11.2	3.16	1.78E-19
TTBK1	tau tubulin kinase 1	5.9	2.26	1.06E-27
ULBP1	UL16 binding protein 1	9.6	3.99	4.90E-23
VWC2	von Willebrand factor C domain containing 2	6.7	2.25	1.81E-11
WISP1	WNT1 inducible signaling pathway protein 1	6.8	2.23	2.26E-18
WNT3A	wingless-type MMTV integration site family, member 3A	8.1	2.72	4.46E-17
XLOC_l2_007928	lincRNA (XLOC_l2_007928), lincRNA [TCONS_l2_00014464]	11.2	2.73	2.55E-16

Meta-analysis showed there were 3156 genes consistently significantly (*q* < 10^−4^) differentially expressed between GC and CC, with 1596 showing higher expression in GC and 1560 in CC. A high concordance was found between the results of our and previous two studies in terms of the direction of expression of differentially expressed genes, as well as the selection of the highest significantly differentially expressed genes. The list of 500 most differentially expressed genes across the three studies is presented in S2 and S3 Tables for GC and CC, respectively.

### Gene ontology and IPA analysis

To determine significantly (p < 0.01) overrepresented biological functions in each cell population the list of differentially expressed genes in our study (524 up-regulated in GC, 126 up-regulated in CC) was subjected to GO analysis. Functions such as immune response, inflammatory response and chemotaxis were among the overrepresented in the GC compartment (Table 3). In the CC compartment protein binding, cell adhesion and multicellular organismal development were among overrepresented (Table 4).

**Table 3:** Top enriched biological functions in GC according to gene ontology analysis. Hyp_c: hypergeometric distribution used for p value calculation.

Items	Items details	Hyp_c
GO:0006954	Inflammatory response	5.00E-09
GO:0006935	Chemotaxis	2.93E-06
GO:0006955	Immune response	5.55E-06

**Table 4:** Top enriched biological functions in CC according to gene ontology analysis. Hyp_c: hypergeometric distribution used for p value calculation.

Items	Items details	Hyp_c
GO:0005515	Protein binding	0.01
GO:0007155	Cell adhesion	0.03
GO:0007275	Multicellular organismal development	0.03

**Fig 2:**
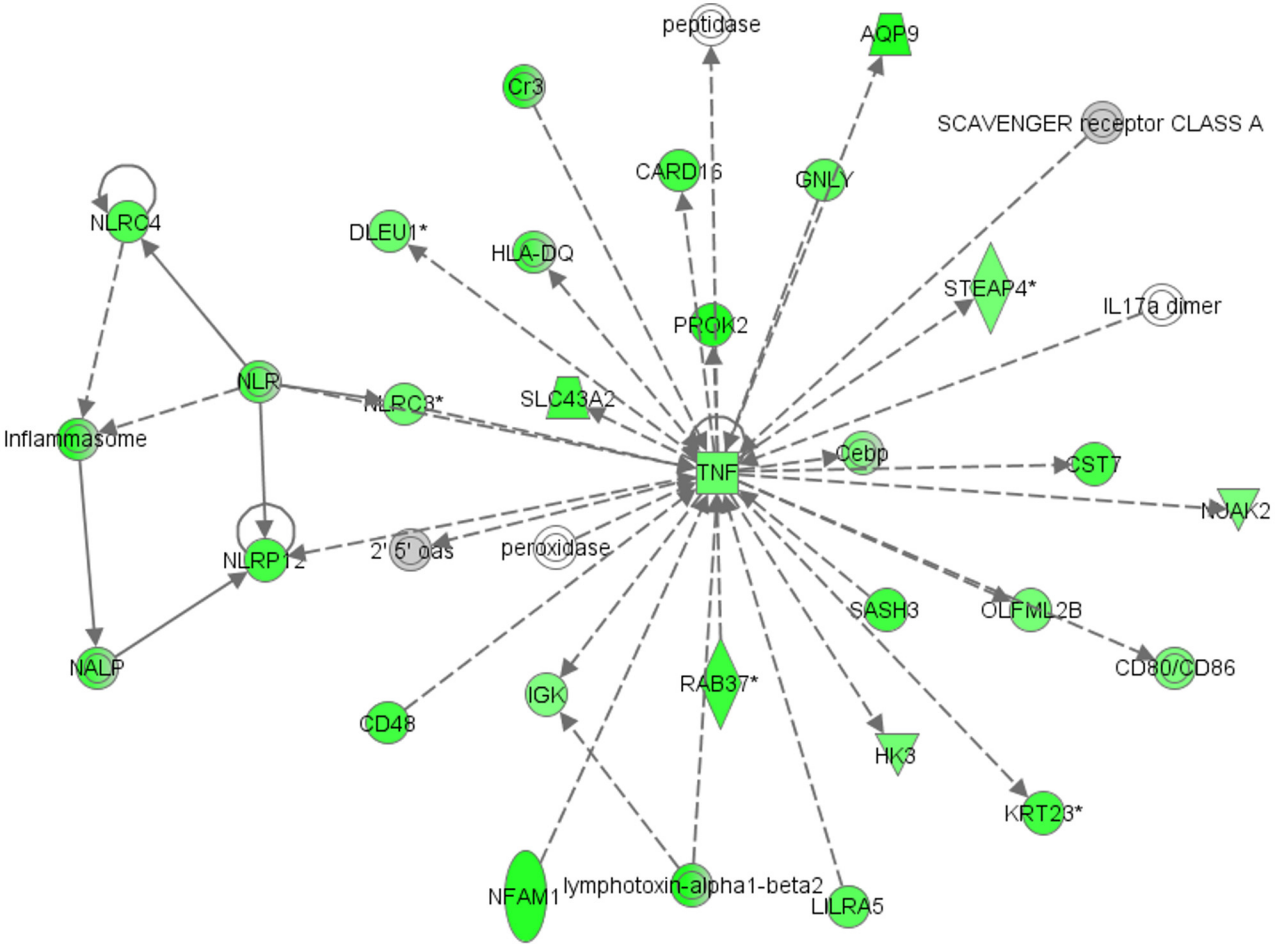
Top network enriched in granulosa cells (Enrichment score: 32) generated with Ingenuity Pathway Analysis (IPA). The network is associated with hematological system development and function, cancer and cellular movement. Up-regulated genes in our study are marked in green colour; the colour intensity of the nodes indicates the degree of up-regulation. Transcripts in grey were not differentially expressed. Genes are represented as nodes, and the biological relationship between two nodes is represented as a line: the plain line indicates direct interaction, the dashed line indicates indirect interaction; the line without arrowhead indicates binding only, the line finishing with a vertical line indicates inhibition; the line with an arrowhead indicates ‘acts on’.

IPA Software was used for network enrichment analysis of differentially expressed genes in each cell subpopulation. Top network enriched in the GC compartment was connected with hematological system development and function, humoral immune response and cellular movement (Enrichment score: 32) (Fig 2). Thirty genes of this network were highly expressed in GC in our study; among them being tumor necrosis factor (*TNF*) gene, which is at the central position of the network.

**Fig 3:**
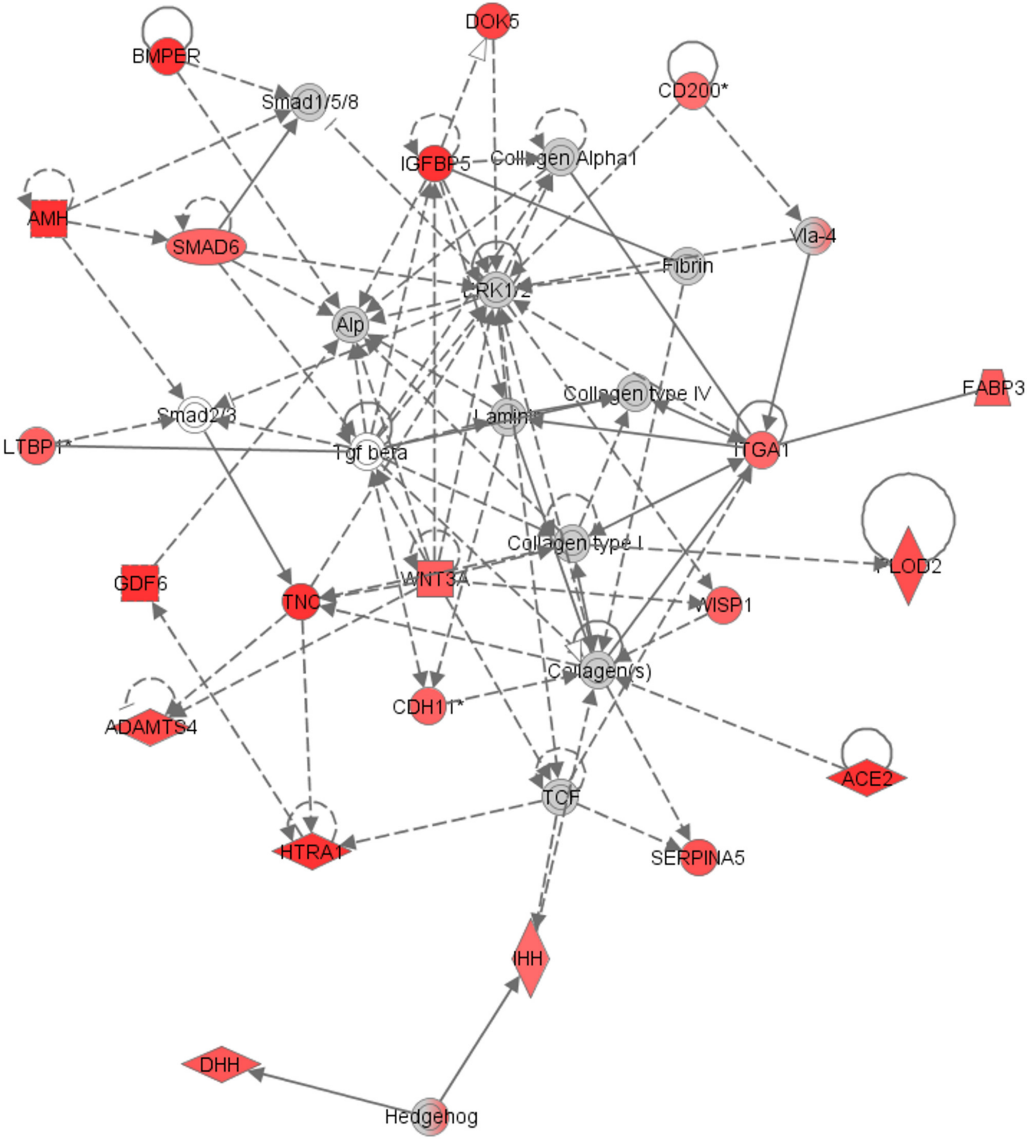
Top network enriched in cumulus cells (Enrichment score: 41) generated with Ingenuity Pathway Analysis (IPA). The network is associated with cellular development, skeletal and muscular system development and function and tissue development. Up-regulated genes in our study are marked in red colour; the colour intensity of the nodes indicates the degree of up-regulation. Transcripts in grey were not differentially expressed. Genes are represented as nodes, and the biological relationship between two nodes is represented as a line: the plain line indicates direct interaction, the dashed line indicates indirect interaction; the line without arrowhead indicates binding only, the line finishing with a vertical line indicates inhibition; the line with an arrowhead indicates ‘acts on’.

In the CC compartment, the top enriched network was connected with cellular development, skeletal and muscular system development and function and tissue development (Enrichment score: 41) (Fig 3). There were 21 genes of this network highly expressed in CC in our study. The network includes insulin-like growth factor binding protein 5 (*IGFBP5*) and tenascin C (*TNC*) genes with functions in extracellular matrix.

### Validation of differential expression by quantitative real-time PCR

**Fig 4:**
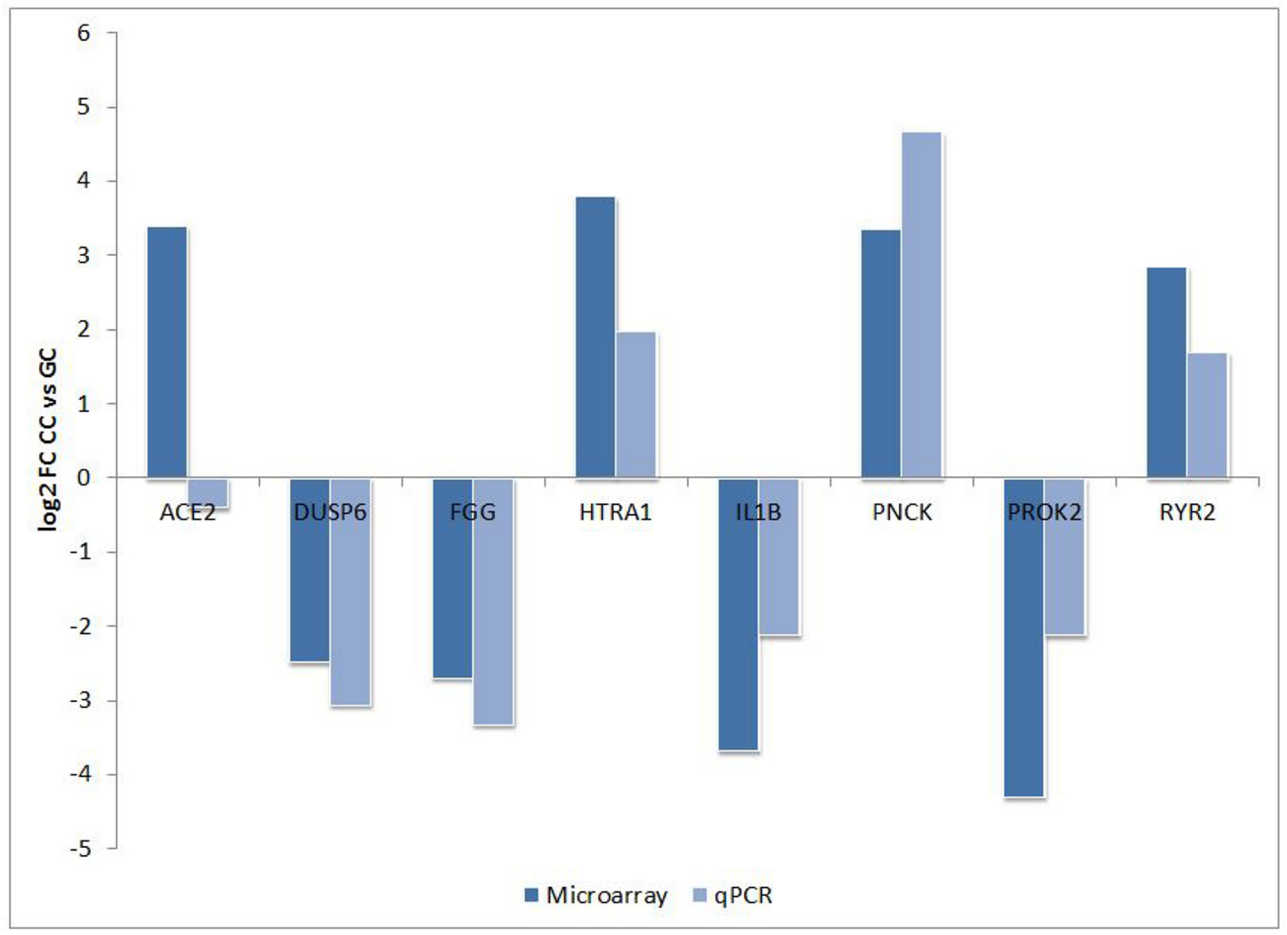
Validation of microarray results by qPCR in additional 19 GC and 19 CC samples, derived from sixteen women. For all genes, except *ACE2*, there was a statistically significant difference in expression between CC and GC. Data are presented as mean log_2_ fold change (FC) between expression in CC and GC. Blue bars represent log_2_ FC as measured by microarray; red bars represent log_2_ FC as measured by qPCR.

**Fig 5:**
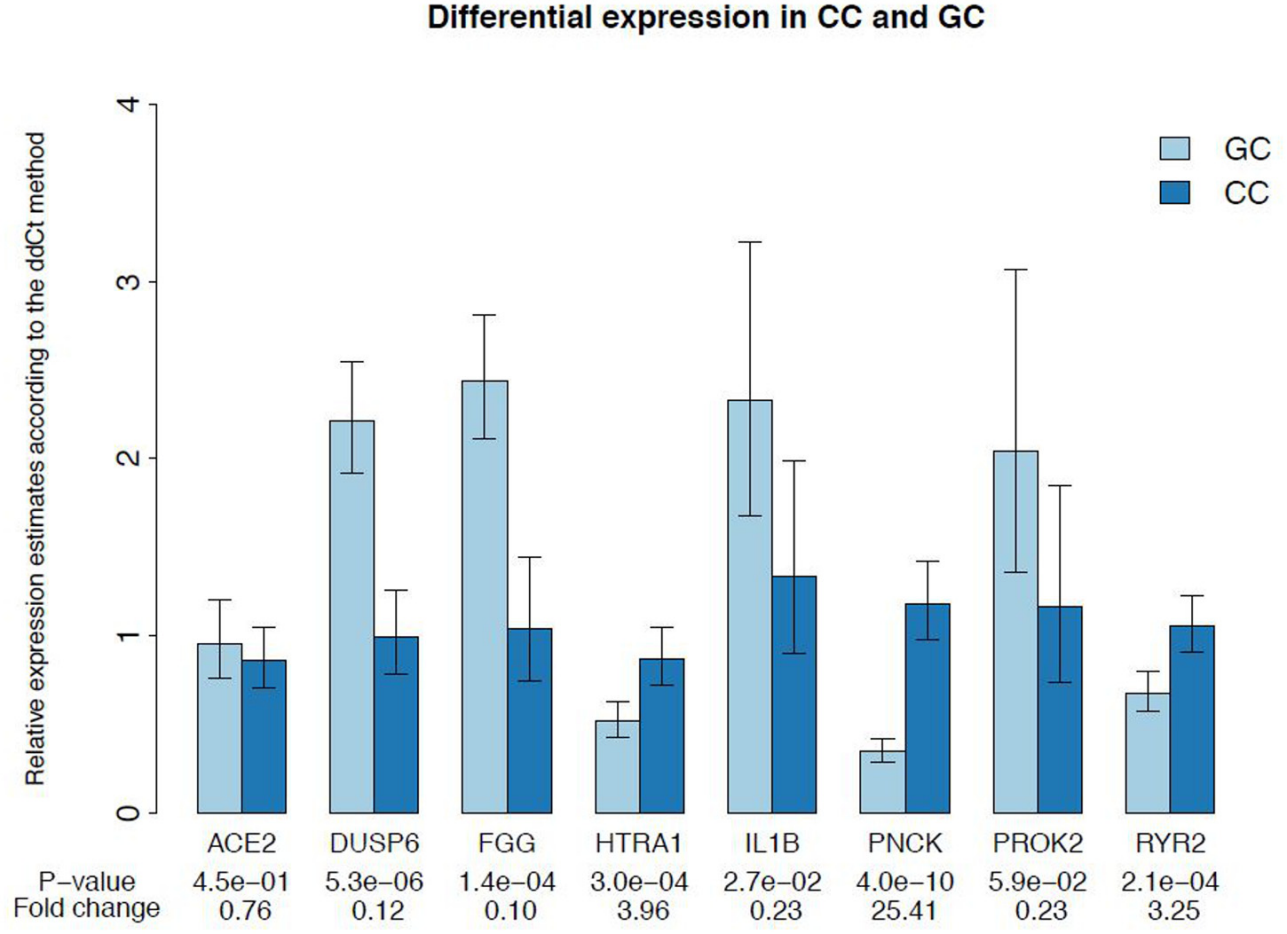
qPCR validation of microarray data. The results represent mean relative mRNA expression of the selected genes ± SEM.

Microarray data from our study were validated using qPCR. Expression levels of 8 genes were checked on 19 individual GC and 19 individual CC samples derived from 16 newly included women. According to the microarray data, genes *DUSP6*, *FGG*, *IL1B* and *PROK2* showed higher expression in GC, whereas *ACE2*, *HTRA1*, *PNCK* and *RYR2* showed higher expression in CC. Results of the qPCR analysis were in agreement with microarray data for 7 out of 8 selected genes (Fig 4). According to qPCR data, the expression of these 7 genes significantly differed between GC and CC samples (Fig 5). For *ACE2* gene, the qPCR analysis failed to confirm the differential expression from the microarray data.

## Discussion

In the present study differential gene expression between human GC and CC surrounding mature oocytes was analysed. Transcriptome profiling of individual human GC and CC samples revealed two distinct cell subpopulations with 7033 genes significantly (*q* < 10^−4^) differentially expressed. Out of these, 650 genes had at least a 2-fold log_2_ difference of expression.

As the number of study samples included in microarray studies is generally much lower than the number of tested variables (genes), testing large number of variables on a small number of study samples can result in false-positive and false-negative detections. This effect can contribute to low reproducibility of results between the studies performed by different research groups [23]. Combining datasets from several experiments where same condition has been investigated can increase the statistical power for detection of biologically relevant genes [24]. For this reason, we decided to perform a meta-analysis in order to maximize the potential for the discovery of consistently differentially expressed genes between human GC and CC. There were 3156 genes that showed consistent significant differential expression between GC and CC after meta-analysis. Among these, there were genes that have not yet been reported to be expressed in human somatic follicular cells like *PROK2* and *PNCK*.

### Differentially expressed genes

The list of significantly differentially expressed genes derived from our study, as well as from the meta-analysis, contained genes that have not yet been described in human GC and CC, like *PROK2* and *PNCK*. qPCR analysis confirmed differential expression of these two genes.

The *PROK2* gene was over-expressed in GC. Prokineticin 1 and 2 (PROK1 and 2) are multifunctional proteins that were first described in the gastrointestinal tract, where they induce contractions of smooth muscle fibres [25]. They exert their effects through G-protein coupled receptors, prokineticin receptor 1 and 2 (*PROKR1* and *PROKR2*) [26]. *PROK2* is mainly expressed in the central nervous system and nonsteroidogenic cells of the testes [27]. It has been established that *PROK2* system is involved in angiogenesis [27], control of circadian rhythms [28] and sexual maturation [29]. *PROK2* was shown to be expressed in the olfactory bulb of mice, where it is involved in neurogenesis and morphogenesis. Homozygous *PROK2* and *PROKR2* knockout mice show hypoplasia of the olfactory bulb [30] and atrophy of the reproductive system (testis, ovary, uterus, vagina, mammary gland) [31]. Defects of the *PROK2* pathway in humans affect the neuroendocrine control of reproduction by causing hypogonadotropic hypogonadism due to the GnRH deficiency, which is caused by a reduction in the number of GnRH neurons in the preoptic region of the hypothalamus [32]. Furthermore, inactivating mutations of *PROK2* and *PROKR2* have been discovered in 2–7% of patients with Kallmann syndrome, who suffer from anosmia and hypogonadotropic hypogonadism [33]. According to the IPA analysis in our study, *PROK2* gene was a part of the top enriched network connected to hematological system development and function, cancer and cellular movement. The development of perifollicular vascularization during folliculogenesis is of fundamental importance for the nourishment of the growing follicle and thus, development of a mature and competent oocyte [34]. Vascular endothelial growth factor (*VEGF*) is an important regulator of this process [35]. In the bovine corpus luteum (CL), *PROK1* stimulates the expression of *VEGF* and this finding implies that it has an indirect role in the process of angiogenesis in the CL [36]. Expression of *PROK2* in GC might imply that this gene also has a role in angiogenesis during folliculogenesis. Furthermore, *PROK2* in the enriched IPA network was indirectly related to the tumor necrosis factor (*TNF*) gene at the central position. During mammalian ovulation, *TNF* causes apoptosis of ovarian surface epithelium and breakdown of the extracellular matrix in the follicle wall [37]. By this the oocyte can be released from the follicle. The connection of *PROK2* with *TNF* might thus indicate that *PROK2* is involved in the process of releasing the cumulus-oocyte complex from the follicle at the time of ovulation.

Another gene that has not been previously described in somatic cells of ovarian follicles and showed higher expression on our set of CC samples and after meta-analysis was *PNCK*. *PNCK* is a calmodulin kinase I isoform [38]. *PNCK* has a role in the murine central nervous system development, as its mRNA and protein are present there during embryonic development [39]. In adult rats, it is also expressed in the breast, uterus, heart and stomach [40]. It has been shown that *PNCK* mRNA over-expression causes epidermal growth factor receptor (EGFR) protein degradation leading to inhibition of EGF-induced mitogen-activated protein kinase (MAPK) activation [41]. After the LH surge, EGF-like factors (amphiregulin, epiregulin, *β*-cellulin) act on *EGFR* to activate *Ras*-MAPK3/1 pathway in GC and CC; this activation is crucial for the CC expansion, final oocyte maturation and GC luteinization [42]. In our study, only CC samples that surrounded mature oocytes were used for analyses; therefore, we presume that *PNCK* expression in CC after LH/hCG triggering oocyte maturation perhaps maintains inactivation of *Ras*-MAPK3/1 pathway, as the effects of this pathway are no longer needed with CC expansion and oocyte maturation being complete.

Among consistently differentially expressed genes that have already been described in human GC and CC there were also *FGG* and *RYR2*. Gene *FGG* that codes fibrinogen was expressed higher in GC. This finding is in accordance with a study of Parrot *et al*. [43], where higher expression of *FGG* was found in equine GC and the presence of FSH increased its expression. Fibrinogen is crucial for blood coagulation and tissue reparation, and its presence in the ovary is probably necessary for restoration of tissue integrity after follicle rupture at ovulation. Gene *RYR2*, a regulator of intracellular Ca++ concentrations, showed higher expression in CC. Positive correlation of *RYR2* expression with expression of genes involved in cell to cell communication in CC has been described [11]. Our findings further support the assumption that intercellular signaling in CC is dependent upon gap junction proteins as well as Ca++ signaling.

Interestingly, we also identified a number of long non-coding RNA (lncRNA) transcripts as differentially expressed, when comparing GC and CC expression profiles. LncRNAs are transcripts of >200 nucleotides without known protein coding function [44]. They play a role in regulating gene expression at the level of chromatin modifications, transcription and post-transcriptional processing [45]. In human CC, differential expression of several lncRNAs was found between mature and germinal vesicle oocytes [46] and between high and poor-quality embryos [47]. These results together with the results of our study show that lncRNAs play a role in the development of oocytes. It is possible that lncRNAs expression participates in driving the differentiation of GC and CC and further studies are required to characterize their biological functions during the process of oocyte maturation.

### Biological functions enriched in GC

The 524 up-regulated genes in GC represent biological functions such as inflammatory and immune response, and these results are consistent with previous studies [10, 11]. Ovulation is considered to be a process similar to inflammation due to the rupture of surface epithelium followed by a healing process [48]. After the release of the oocyte from the ovary the coagulation cascade is activated through invasion of leucocytes and cytokine release, that are needed for tissue healing [49]. Among the genes involved in the inflammatory process that were significantly up-regulated in GC in our study was *IL1B*. IL1 system is composed of ligands *IL1A* and *IL1B* [50], receptors *IL1R1* and *IL1R2* [51] and a receptor antagonist *IL1RA* [52]. In human cultured GC *IL1B* was shown to regulate plasminogen activator activity and steroidogenesis [53]. In our study *IL1B*, *IL1RA* and *IL1R2* were up-regulated in GC, *IL1A* expression was undetectable and *IL1R1* was up-regulated in CC. These findings are in accordance with the study of Martoriati and Gérard [54], where equine GC showed the same expression of IL1 system genes. They confirm the proposed involvement of the IL1 system in the events regulating oocyte maturation and ovulation [55] (Gérard *et al*., 2004). The higher expression of *IL1R1* in CC probably indicates that *IL1B* exerts its effects on CC through this receptor. In the present study, *IL6* and *IL8* were also up-regulated in GC. This finding is in accordance with previous studies, where it has been established that *IL1* induces expression of pro-inflammatory genes (*IL6*, *IL8*) at the site of the follicle rupture [56]. Cytokines IL6 and IL8 induce cellular proliferation which is needed for the healing of the ovarian surface epithelium [57]. Furthermore, *IL6* has an important role in ovulation process as it is involved in regulation of meitoic maturation, CC expansion and CL formation [58].

The group of 'toll-like' receptors (*TLR1*,*2*, *4*, *6*, *7*, *8*), that has a role in the innate immune response in somatic cells of ovarian follicles [59], was found to be up-regulated in GC in our study. Our results are in line with a previous study that compared transcriptomes of GC and CC [10]. In mouse CC, *TLR* genes activate the expression of several signalling molecules (*IL6*, *PTGS2*, *PTX3*) that have a known role in CC expansion [60], cumulus-oocyte complex oviductal migration [61] and fertilization [62]. In hen GC, treatment with LH led to an increase in *TLR*'s mRNA in pre-ovulatory follicles [59]. In the study of Woods *et al*. [63] it was shown that *TLR* signaling pathway influences hen GC steroidogenesis and apoptosis in relation to follicle size. Additionally, it has been suggested that *TLR*'s function as a detection system to initiate tissue regeneration after injury [64]. According to these findings, we may presume that *TLR* signaling pathway also modulates human folliculogenesis. Furthermore, the high expression of *TLR*'s in GC of pre-ovulatory follicles might support the suggestion that they are important in the healing process at the sight of the follicle rupture after ovulation. Additional studies are needed to determine the effects of *TLR* mediated signaling in GC during folliculogenesis.

### Biological functions enriched in CC

The 126 significantly up-regulated genes in CC represent biological functions as multicellular organismal development and cell differentiation. Genes *IGFBP5* and *TNC* were among the genes involved in signal transduction. IGFBP5 is a binding protein for insulin-like growth factors 1 and 2 (IGF1, IGF2), that are known to stimulate extracellular matrix production [65] and regulate steroidogenesis [66] during folliculogenesis. IGFBP proteins control the bioactivity of IGFs and controlled access to IGF1 is crucial for the proper coordination of early oocyte and follicular development [67]. In the present study, *IGFBP5* expression was increased in CC and *IGF1* expression was increased in GC. These results are in line with the study of Ingman *et al*. [68] where treatment of CC culture media with IGF1 increased extracellular matrix IGFBP5 by 2.5-fold. Our results probably indicate that in pre-ovulatory follicles, bioavailability of IGF1 protein derived from GC is regulated by *IGFBP5* expression in CC.

*TNC* is an important glycoprotein of the extracellular matrix [69]. It is involved in cellular functions as adhesion, migration, embryonic development, wound healing and tumour metastasis [70]. It was shown to be overexpressed in the stroma of malignant ovarian tumours and it was proposed to be involved in the process of tumour invasion [71]. In ovarian cancer cell line expressions of transforming growth factor beta 1 (*TGFB1*) and *TNC* are significantly related [72]. Furthermore, *TGFB1* induces secretion of *TNC* from ovarian fibroblasts [68]. During folliculogenesis *TGFB1* enables CC hyaluronic acid production and expansion, regulates CC steroidogenesis and promotes GC proliferation [73, 74, 75]. *TNC* was significantly higher expressed in CC after our microarray analysis as well as after the meta-analysis. Over-expression of *TNC* in CC might thus indicate that *TNC* is one of the genes through which *TGFB1* exerts its effects during the process of oocyte maturation. Evidently, if this process is pathological, it is related to ovarian tumour invasion. The precise role of *TNC* in ovarian folliculogenesis and the possible relation with *TGFB1* will need to be determined in future studies.

In conclusion, with this study we upgraded the existing data on differentially expressed genes between GC and CC by description of *PROK2* and *PNCK* genes whose expression has, to the best of our knowledge, not yet been reported in human GC and CC. These genes could be involved in any number of the processes regulating folliculogenesis. Further research is needed to elucidate their potential physiological roles. Pathway enrichment and IPA analyses suggested that GC are involved in angiogenesis and ovarian healing process after follicle rupture, whereas pathways enriched in CC are connected with intercellular communication and extracellular matrix formation. Identification of genes and pathways that contribute to the development of mature and competent oocytes has the potential to improve oocyte *in vitro* maturation procedures.

## Supporting Information

S1 PRISMA Checklist (DOC)

S1 PRISMA Flow Chart (DOC)

S1 Table Full list of differentially expressed genes between GC and CC. (DOC)

S2 Table Top 500 differentially expressed genes with higher expression in GC. (DOCX)

S3 Table Top 500 differentially expressed genes with higher expression in CC. (DOCX)
