# Supplementary material for: Transcriptomic Analysis and Meta-Analysis of Human Granulosa and Cumulus Cells
Source: PLoS One. 2015 Aug 27;10(8):e0136473. doi: 10.1371/journal.pone.0136473 (PMC4552299; doi:10.1371/journal.pone.0136473)
Supplement: S1 PRISMA Flow Chart — (DOC) [file pone.0136473.s002.doc]

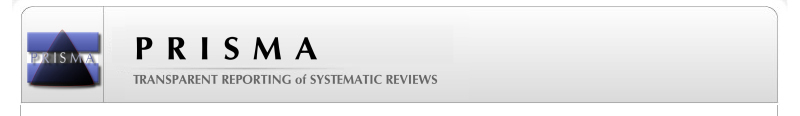
**PRISMA 2009 Flow Diagram**

**Screening**

**Included**

**Eligibility**

**Identification**

Records identified through database searching
(n = 2 )

Additional records identified through other sources
(n = 0 )

Records after duplicates removed
(n = )

Records screened
(n = )

Records excluded
(n = 0 )

Full-text articles assessed for eligibility
(n = 2 )

Full-text articles excluded, with reasons
(n = 0 )

Studies included in qualitative synthesis
(n = 2 )

Studies included in quantitative synthesis (meta-analysis)
(n = 2 )
