# Supplementary material for: Transcriptomic Analysis and Meta-Analysis of Human Granulosa and Cumulus Cells
Source: PLoS One. 2015 Aug 27;10(8):e0136473. doi: 10.1371/journal.pone.0136473 (PMC4552299; doi:10.1371/journal.pone.0136473)
Supplement: S1 Table — (DOC) [file pone.0136473.s003.doc]

**S1 Table: 650** differentially expressed genes between CC and GC.

| **Gene symbol** | **logFC(CC/GC)** | **adjusted P value** |
| --- | --- | --- |
| A2M | -2.02 | 6.68E-11 |
| ABTB1 | -2.59 | 6.97E-13 |
| ACE2 | 3.39 | 1.33E-17 |
| ACOXL | 3.13 | 1.97E-16 |
| ACP5 | -2.30 | 5.47E-11 |
| ACRBP | -2.03 | 1.62E-12 |
| ACSM5 | -2.44 | 1.26E-10 |
| ACTA1 | 2.72 | 1.24E-17 |
| ADAM8 | -3.30 | 2.40E-18 |
| ADAMTS4 | 2.58 | 3.22E-19 |
| ADAP2 | -2.42 | 7.09E-14 |
| AHSP | -2.99 | 3.09E-13 |
| AIF1 | -3.44 | 6.99E-14 |
| AK021933 | -2.80 | 5.74E-13 |
| AK092494 | -2.82 | 2.84E-12 |
| AK098147 | -2.69 | 3.75E-13 |
| ALAS2 | -3.57 | 2.76E-14 |
| ALPK2 | 2.37 | 4.46E-17 |
| AMH | 3.16 | 9.13E-18 |
| AMICA1 | -2.06 | 9.66E-15 |
| ANKRD33 | -3.16 | 3.88E-13 |
| ANPEP | -2.54 | 1.27E-09 |
| ANXA1 | -2.21 | 9.77E-10 |
| ANXA3 | -2.31 | 2.32E-17 |
| AOAH | -2.51 | 1.63E-11 |
| APBA1 | 2.02 | 2.49E-13 |
| APBB1IP | -3.57 | 9.89E-15 |
| APOBEC3A | -3.30 | 9.42E-14 |
| APOBEC3B | -2.17 | 4.95E-12 |
| APOBR | -3.31 | 8.27E-15 |
| AQP9 | -3.62 | 4.10E-16 |
| ARG2 | 2.12 | 2.24E-11 |
| ARHGAP15 | -2.55 | 7.02E-10 |
| ARHGAP25 | -2.96 | 2.70E-16 |
| ARHGAP27 | -2.63 | 2.74E-11 |
| ARHGAP30 | -3.59 | 7.09E-15 |
| ARHGAP6 | 2.15 | 1.53E-12 |
| ARHGAP9 | -2.61 | 1.03E-10 |
| ARHGDIB | -3.59 | 1.78E-16 |
| ARRB2 | -2.06 | 1.58E-17 |
| BCL2A1 | -3.52 | 1.50E-12 |
| BEX1 | 3.24 | 6.30E-25 |
| BIN2 | -2.67 | 4.04E-13 |
| BLVRB | -2.03 | 6.00E-13 |
| BMPER | 3.19 | 1.48E-22 |
| BST1 | -2.08 | 4.81E-10 |
| BTG2 | -2.03 | 8.42E-09 |
| BTK | -2.13 | 8.73E-15 |
| BTNL8 | -2.56 | 9.51E-15 |
| BUB1 | 2.27 | 1.24E-12 |
| C10orf54 | -2.52 | 4.58E-11 |
| C13orf15 | -2.08 | 1.59E-07 |
| C16orf54 | -4.03 | 4.33E-16 |
| C17orf109 | -2.10 | 3.91E-07 |
| C17orf87 | -2.17 | 5.95E-11 |
| C19orf59 | -2.82 | 4.85E-13 |
| C1orf141 | 2.45 | 3.58E-13 |
| C1orf162 | -2.67 | 1.16E-09 |
| C20orf103 | 2.04 | 7.87E-22 |
| C20orf108 | -2.60 | 7.54E-10 |
| C2CD4B | 2.64 | 2.39E-17 |
| C5AR1 | -4.03 | 1.27E-17 |
| C9orf173 | -2.09 | 2.58E-07 |
| CA1 | -2.92 | 1.46E-12 |
| CACNA1C | 2.34 | 3.52E-14 |
| CAMP | -3.26 | 3.01E-17 |
| CARD16 | -3.00 | 6.31E-15 |
| CARD17 | -2.81 | 6.28E-12 |
| CARD9 | -2.32 | 1.15E-15 |
| CASP1 | -3.48 | 7.57E-17 |
| CBLN1 | 2.51 | 1.26E-18 |
| CCDC3 | 2.20 | 1.13E-19 |
| CCDC88B | -2.61 | 4.49E-10 |
| CCL2 | -2.45 | 1.86E-08 |
| CCL3 | -3.22 | 3.81E-15 |
| CCL3L3 | -2.12 | 4.52E-09 |
| CCL4 | -3.16 | 2.16E-17 |
| CCL5 | -3.48 | 9.49E-18 |
| CCR1 | -3.45 | 1.65E-15 |
| CCR2 | -2.44 | 4.52E-15 |
| CCR5 | -2.39 | 2.29E-13 |
| CCR7 | -3.48 | 4.10E-15 |
| CD14 | -2.26 | 5.16E-09 |
| CD163 | -2.54 | 1.04E-09 |
| CD2 | -3.12 | 5.72E-12 |
| CD200 | 2.04 | 2.54E-08 |
| CD247 | -3.28 | 3.00E-12 |
| CD27 | -2.54 | 1.21E-09 |
| CD300A | -3.21 | 8.07E-15 |
| CD300LF | -2.50 | 9.51E-14 |
| CD33 | -2.87 | 4.80E-15 |
| CD36 | -2.17 | 1.19E-09 |
| CD37 | -2.91 | 1.12E-11 |
| CD3G | -2.22 | 1.79E-12 |
| CD48 | -3.05 | 1.65E-10 |
| CD5 | -2.20 | 1.28E-07 |
| CD52 | -3.32 | 4.32E-11 |
| CD53 | -3.26 | 2.32E-13 |
| CD6 | -2.09 | 2.87E-07 |
| CD74 | -2.43 | 8.25E-12 |
| CD86 | -2.08 | 9.43E-13 |
| CD8A | -2.39 | 2.28E-14 |
| CD93 | -2.56 | 3.10E-11 |
| CD97 | -3.49 | 4.91E-14 |
| CDH11 | 2.24 | 1.07E-19 |
| CEBPA | -2.16 | 3.02E-12 |
| CFD | -3.14 | 1.47E-13 |
| CHGA | 2.57 | 3.33E-13 |
| CHGB | 2.24 | 3.95E-19 |
| CHST15 | -3.00 | 1.77E-12 |
| CIB4 | 2.87 | 5.64E-20 |
| CILP | 3.10 | 6.85E-13 |
| CLEC10A | -2.73 | 1.95E-10 |
| CLEC4A | -2.72 | 4.82E-13 |
| CLEC4D | -2.24 | 5.54E-10 |
| CLEC7A | -2.67 | 7.52E-14 |
| CLMP | 2.18 | 8.07E-22 |
| CLSTN2 | 2.26 | 4.30E-14 |
| CMKLR1 | -2.53 | 1.29E-08 |
| CMTM2 | -3.51 | 1.07E-13 |
| COL6A2 | 2.01 | 6.40E-12 |
| CORO1A | -2.37 | 1.25E-08 |
| CORO2A | 2.61 | 2.44E-22 |
| CPVL | -2.38 | 1.88E-12 |
| CREB5 | -2.84 | 9.10E-11 |
| CRHBP | 2.08 | 7.33E-13 |
| CRYAA | -2.19 | 1.03E-08 |
| CSF2RA | -2.19 | 4.04E-14 |
| CSF3R | -2.39 | 7.91E-09 |
| CST7 | -2.94 | 7.08E-11 |
| CSTA | -2.41 | 7.31E-16 |
| CTSK | 2.78 | 1.32E-13 |
| CX3CR1 | -3.50 | 2.57E-13 |
| CXCL2 | -2.72 | 1.75E-16 |
| CXCR1 | -2.73 | 2.67E-14 |
| CXCR2 | -3.18 | 8.59E-13 |
| CXorf21 | -2.21 | 3.88E-13 |
| CYBB | -3.02 | 3.96E-12 |
| CYP19A1 | 3.25 | 3.11E-18 |
| CYTH4 | -3.28 | 3.99E-15 |
| CYTIP | -3.40 | 1.68E-13 |
| DAPK2 | -2.09 | 3.37E-10 |
| DAPL1 | 3.51 | 1.78E-19 |
| DAPP1 | -2.54 | 3.08E-10 |
| DB335107 | -3.17 | 5.33E-18 |
| DCLK1 | 2.07 | 1.21E-14 |
| DEFA3 | -4.02 | 2.00E-13 |
| DHH | 2.40 | 2.71E-14 |
| DKFZp451A211 | 2.46 | 2.44E-18 |
| DLX5 | 2.21 | 2.42E-16 |
| DNM3 | 2.14 | 7.55E-15 |
| DOCK11 | -2.39 | 8.31E-11 |
| DOCK8 | -2.82 | 1.02E-14 |
| DOK3 | -2.79 | 1.18E-12 |
| DOK5 | 2.65 | 4.18E-13 |
| DPEP2 | -2.63 | 4.49E-11 |
| DPYSL4 | 2.34 | 7.06E-18 |
| DUOXA2 | 2.60 | 2.75E-12 |
| DUSP2 | -2.01 | 5.96E-10 |
| DUSP6 | -2.48 | 2.45E-11 |
| DYSF | -2.07 | 2.58E-08 |
| E2F7 | 3.44 | 2.36E-23 |
| EGR2 | -2.66 | 1.31E-16 |
| ELMOD3 | -2.28 | 1.54E-12 |
| EMB | -2.62 | 7.45E-14 |
| EMR2 | -2.32 | 2.50E-15 |
| EMR3 | -2.98 | 5.44E-15 |
| ENST00000381524 | -2.69 | 4.82E-12 |
| ENST00000390252 | -2.02 | 2.13E-09 |
| ENST00000390477 | -2.40 | 4.41E-09 |
| ENST00000390547 | -3.23 | 1.48E-14 |
| ENST00000390551 | -2.10 | 5.41E-10 |
| ENST00000477036 | -2.41 | 2.71E-09 |
| EPB42 | -3.09 | 4.41E-14 |
| EPB49 | -3.11 | 8.23E-17 |
| EPHB1 | 2.41 | 5.41E-18 |
| EVI2A | -2.74 | 8.57E-14 |
| EVI2B | -3.73 | 1.16E-16 |
| F13A1 | -2.37 | 2.95E-15 |
| F3 | 2.34 | 1.81E-11 |
| FABP3 | 2.31 | 4.59E-12 |
| FAM110C | 2.46 | 4.79E-15 |
| FAM150B | 2.93 | 1.35E-12 |
| FAM181A | -2.07 | 2.41E-10 |
| FAM189A1 | 2.26 | 2.26E-14 |
| FAM46C | -2.01 | 3.52E-14 |
| FAM65B | -3.37 | 1.42E-10 |
| FBRS | -2.54 | 5.11E-13 |
| FCAR | -3.37 | 4.21E-18 |
| FCER1A | -2.83 | 8.96E-15 |
| FCGR1B | -2.19 | 9.73E-12 |
| FCGR2A | -3.50 | 2.86E-15 |
| FCGR2B | -2.45 | 1.68E-12 |
| FCGR2C | -2.98 | 5.96E-12 |
| FCN1 | -2.85 | 1.69E-15 |
| FERMT3 | -2.29 | 1.49E-12 |
| FFAR2 | -2.33 | 1.02E-12 |
| FGD3 | -3.38 | 5.52E-14 |
| FGF11 | 2.42 | 7.90E-11 |
| FGG | -2.69 | 1.58E-18 |
| FGL2 | -2.28 | 5.86E-14 |
| FGR | -2.82 | 1.19E-11 |
| FLI1 | -3.10 | 4.40E-15 |
| FLRT3 | 2.00 | 1.35E-07 |
| FN1 | 2.52 | 1.14E-15 |
| FOLR3 | -2.11 | 4.93E-13 |
| FOS | -2.47 | 1.57E-13 |
| FOSB | -3.65 | 3.37E-17 |
| FOXG1 | 3.30 | 9.22E-15 |
| FPR2 | -3.40 | 6.30E-14 |
| FPR3 | -2.58 | 6.24E-12 |
| FYB | -3.11 | 4.77E-15 |
| GABBR2 | 2.47 | 1.97E-16 |
| GABRP | -3.85 | 6.37E-15 |
| GAL | 2.93 | 2.55E-16 |
| GAP43 | 2.36 | 1.36E-15 |
| GBP5 | -2.72 | 7.56E-11 |
| GCA | -2.74 | 5.37E-13 |
| GDF6 | 3.36 | 5.64E-20 |
| GIMAP1 | -3.13 | 4.35E-13 |
| GIMAP2 | -2.17 | 4.18E-14 |
| GIMAP4 | -3.27 | 1.40E-16 |
| GIMAP5 | -2.68 | 7.77E-10 |
| GIMAP6 | -2.65 | 3.44E-13 |
| GIMAP7 | -3.03 | 2.23E-12 |
| GIMAP8 | -2.15 | 7.96E-18 |
| GJA5 | 2.36 | 2.51E-19 |
| GLT1D1 | -3.31 | 1.44E-14 |
| GMFG | -3.72 | 1.80E-13 |
| GMPR | -2.14 | 2.54E-14 |
| GNLY | -2.61 | 8.00E-14 |
| GPNMB | -2.13 | 2.82E-09 |
| GPR158 | 2.01 | 4.05E-09 |
| GPR179 | -2.06 | 1.70E-14 |
| GPR183 | -2.64 | 2.83E-14 |
| GPR56 | 2.07 | 1.12E-13 |
| GPR65 | -2.38 | 1.27E-15 |
| GPR88 | -2.62 | 4.69E-16 |
| GPRC5B | 2.18 | 1.13E-12 |
| GPSM3 | -2.06 | 2.42E-09 |
| GRIK3 | 2.76 | 2.17E-18 |
| GSTA5 | 2.04 | 2.51E-12 |
| GSTM3 | 2.04 | 2.09E-07 |
| GYPC | -2.37 | 1.74E-12 |
| GZMA | -3.71 | 4.86E-15 |
| GZMB | -3.46 | 8.10E-13 |
| GZMH | -3.14 | 1.08E-15 |
| GZMK | -2.20 | 6.32E-10 |
| HBA2 | -4.90 | 2.77E-14 |
| HBEGF | -2.10 | 4.50E-14 |
| HBM | -3.57 | 1.78E-15 |
| HBQ1 | -4.11 | 1.87E-15 |
| HCAR3 | -3.42 | 9.50E-09 |
| HCK | -3.26 | 8.87E-17 |
| HCLS1 | -3.74 | 1.17E-12 |
| HCST | -2.38 | 8.77E-12 |
| HEMGN | -2.90 | 2.06E-15 |
| HIGD2B | -2.22 | 5.61E-13 |
| HK3 | -2.25 | 9.10E-10 |
| HLA-DMA | -2.34 | 2.48E-10 |
| HLA-DMB | -2.76 | 7.82E-10 |
| HLA-DPA1 | -2.91 | 1.80E-12 |
| HLA-DPB1 | -2.48 | 9.31E-12 |
| HLA-DPB2 | -2.22 | 2.25E-10 |
| HLA-DQA1 | -2.75 | 7.88E-14 |
| HLA-DQA2 | -2.99 | 4.19E-14 |
| HLA-DRA | -2.22 | 1.50E-11 |
| HLA-F | -2.23 | 7.81E-10 |
| HMBOX1 | -2.10 | 4.64E-12 |
| HMHA1 | -2.42 | 9.90E-14 |
| HSH2D | -2.67 | 8.61E-16 |
| HSPA6 | -3.02 | 1.12E-13 |
| HTRA1 | 3.79 | 8.97E-23 |
| ICAM2 | -2.41 | 2.01E-12 |
| ICAM3 | -2.35 | 2.31E-19 |
| IER2 | -2.58 | 1.15E-09 |
| IFI30 | -2.87 | 4.34E-11 |
| IFIT1 | -2.39 | 2.18E-07 |
| IFIT1B | -2.69 | 1.64E-09 |
| IFIT2 | -3.09 | 8.55E-13 |
| IGFBP5 | 3.54 | 3.15E-26 |
| IGSF6 | -2.87 | 2.63E-16 |
| IHH | 2.12 | 2.50E-19 |
| IKZF1 | -2.34 | 1.38E-10 |
| IL10RA | -3.08 | 9.44E-12 |
| IL16 | -2.55 | 1.18E-12 |
| IL18RAP | -3.43 | 1.18E-11 |
| IL1B | -3.68 | 6.33E-16 |
| IL1R2 | -2.02 | 7.64E-07 |
| IL1RN | -3.06 | 2.71E-18 |
| IL2RB | -3.31 | 1.62E-11 |
| IL2RG | -2.25 | 1.58E-09 |
| IL6R | -2.06 | 1.18E-09 |
| IL8 | -2.53 | 4.64E-15 |
| INPP5D | -3.67 | 4.64E-15 |
| IRF1 | -2.18 | 9.33E-12 |
| IRF8 | -3.04 | 2.53E-14 |
| ISM1 | 2.56 | 6.25E-20 |
| ITGA1 | 2.15 | 6.59E-13 |
| ITGAL | -3.27 | 6.98E-13 |
| ITGAM | -3.18 | 2.74E-18 |
| ITGAX | -2.92 | 1.15E-12 |
| ITGB2 | -3.68 | 6.60E-14 |
| ITLN1 | -2.90 | 8.33E-19 |
| KCNE3 | -2.81 | 9.98E-12 |
| KCNJ15 | -2.61 | 6.96E-14 |
| KCNJ2 | -2.46 | 3.72E-12 |
| KCNK3 | 2.67 | 2.14E-14 |
| KCNQ3 | -2.04 | 1.82E-14 |
| KCNT1 | 2.25 | 1.84E-18 |
| KIAA0226L | -2.36 | 1.16E-14 |
| KIR2DL4 | -2.49 | 2.58E-11 |
| KLF2 | -4.05 | 1.54E-18 |
| KLRB1 | -3.43 | 1.82E-17 |
| KRT1 | -3.43 | 1.71E-13 |
| KRT23 | -3.03 | 2.20E-14 |
| KRTAP13-1 | 2.84 | 4.18E-18 |
| KRTAP13-2 | 2.57 | 1.66E-17 |
| LAIR1 | -2.91 | 3.02E-17 |
| LALBA | -2.53 | 7.79E-13 |
| LAPTM5 | -3.40 | 2.19E-15 |
| LAT2 | -2.97 | 1.20E-13 |
| LCK | -2.34 | 2.23E-11 |
| LCP1 | -3.43 | 2.28E-13 |
| LCP2 | -2.57 | 6.30E-15 |
| LEFTY2 | 2.41 | 5.27E-15 |
| LFNG | -2.75 | 3.35E-08 |
| LGALS2 | -2.83 | 3.34E-12 |
| LGR6 | -2.02 | 2.79E-11 |
| LILRA2 | -2.24 | 6.05E-13 |
| LILRA3 | -2.39 | 3.93E-14 |
| LILRA4 | -2.19 | 1.74E-11 |
| LILRA5 | -2.63 | 1.74E-13 |
| LILRA6 | -2.44 | 4.25E-14 |
| LILRB1 | -2.33 | 7.12E-13 |
| LILRB2 | -2.17 | 6.93E-11 |
| LILRB3 | -2.28 | 2.51E-09 |
| LILRB4 | -2.55 | 2.51E-10 |
| LIMS2 | 2.54 | 2.14E-15 |
| LINC00239 | -2.01 | 1.92E-14 |
| LINC00340 | -2.15 | 2.05E-11 |
| LINC00341 | -2.21 | 1.91E-10 |
| lincRNA:chr1:1100287-1108062_F | -2.85 | 3.72E-12 |
| lincRNA:chr1:181063077-181073127_R | -2.97 | 7.64E-12 |
| lincRNA:chr1:181309852-181429627_F | -3.17 | 1.07E-12 |
| lincRNA:chr1:181309852-181429627_R | -2.72 | 3.14E-11 |
| lincRNA:chr1:205404902-205417627_F | -3.39 | 8.67E-16 |
| lincRNA:chr1:24033913-24039563_F | -2.10 | 6.44E-09 |
| lincRNA:chr1:86062087-86107987_R | -3.04 | 3.00E-14 |
| lincRNA:chr1:93796837-93806487_F | -2.61 | 8.64E-15 |
| lincRNA:chr10:17250419-17261819_F | -3.55 | 4.72E-16 |
| lincRNA:chr11:88105752-88140214_F | -2.29 | 1.12E-14 |
| lincRNA:chr12:130608397-130621372_R | -2.12 | 1.02E-15 |
| lincRNA:chr12:5377839-5428564_R | -2.07 | 1.35E-13 |
| lincRNA:chr12:92517824-92518470_R | -2.27 | 2.29E-10 |
| lincRNA:chr12:96953169-96992769_F | -2.17 | 3.10E-10 |
| lincRNA:chr13:101223049-101236424_R | -2.41 | 1.60E-12 |
| lincRNA:chr13:33888375-33923550_R | -2.01 | 1.49E-10 |
| lincRNA:chr13:50903769-50975502_F | -2.32 | 3.94E-13 |
| lincRNA:chr14:61529972-61553247_F | -2.03 | 4.65E-07 |
| lincRNA:chr15:52209683-52223508_R | -3.38 | 6.18E-14 |
| lincRNA:chr16:85205424-85218749_F | -2.31 | 5.15E-14 |
| lincRNA:chr17:29887612-29925137_F | -3.72 | 3.31E-14 |
| lincRNA:chr17:73598283-73599500_F | -3.74 | 8.00E-14 |
| lincRNA:chr18:36967212-37153620_R | -2.07 | 1.57E-11 |
| lincRNA:chr19:52588083-52597524_F | -2.17 | 1.12E-18 |
| lincRNA:chr2:114566880-114619605_F | -2.81 | 2.37E-13 |
| lincRNA:chr2:121292955-121305755_F | -2.49 | 2.40E-14 |
| lincRNA:chr2:12453499-12466124_R | -2.39 | 1.17E-13 |
| lincRNA:chr2:130194980-130200205_R | -2.41 | 4.88E-10 |
| lincRNA:chr2:200984605-201020245_F | -2.10 | 2.15E-09 |
| lincRNA:chr2:217381355-217413480_F | -2.25 | 1.51E-13 |
| lincRNA:chr2:37776096-37862046_F | -2.56 | 4.61E-13 |
| lincRNA:chr21:39601874-39613891_F | -2.45 | 2.03E-10 |
| lincRNA:chr22:18441799-18444050_R | -2.69 | 6.61E-12 |
| lincRNA:chr22:46451236-46516536_R | -2.61 | 1.24E-10 |
| lincRNA:chr3:139141917-139163444_R | -2.93 | 4.24E-15 |
| lincRNA:chr3:62935685-63116160_F | -2.23 | 2.39E-11 |
| lincRNA:chr4:23771777-23778877_R | -2.17 | 1.25E-15 |
| lincRNA:chr4:3676927-3688227_F | -2.02 | 6.18E-13 |
| lincRNA:chr4:6664099-6683749_F | -2.02 | 9.49E-11 |
| lincRNA:chr4:769425-775573_F | -3.76 | 1.61E-16 |
| lincRNA:chr5:12625075-12747025_F | -3.99 | 4.90E-15 |
| lincRNA:chr6:114722782-114743483_F | -2.20 | 1.07E-11 |
| lincRNA:chr6:158658787-158724862_F | -2.15 | 1.44E-10 |
| lincRNA:chr6:56715896-56716656_R | -3.00 | 5.47E-16 |
| lincRNA:chr6:75996030-76001430_R | -2.69 | 5.30E-13 |
| lincRNA:chr7:22666693-22667114_R | -2.84 | 4.93E-10 |
| lincRNA:chr8:124456094-124469294_R | -2.35 | 8.38E-10 |
| lincRNA:chr8:32772708-32913758_F | -2.19 | 6.95E-11 |
| lincRNA:chr9:2739400-2746875_F | -2.06 | 3.56E-09 |
| lincRNA:chrX:107264119-107280819_F | -2.12 | 1.07E-11 |
| LITAF | -2.99 | 8.77E-14 |
| LMO2 | -3.15 | 1.69E-12 |
| LOC100128348 | -3.25 | 3.62E-13 |
| LOC100128714 | -2.40 | 2.81E-05 |
| LOC100129931 | -2.11 | 1.12E-12 |
| LOC100130920 | -2.80 | 2.17E-12 |
| LOC100133286 | -3.29 | 1.71E-16 |
| LOC100288292 | -2.53 | 2.55E-10 |
| LOC100506189 | 2.43 | 1.24E-16 |
| LOC100508384 | -2.50 | 7.81E-14 |
| LOC100652730 | -3.22 | 2.42E-19 |
| LOC146336 | -2.54 | 1.91E-15 |
| LRAT | 2.79 | 7.96E-18 |
| LRG1 | -2.56 | 8.60E-12 |
| LRMP | -2.81 | 9.28E-16 |
| LRRC25 | -3.19 | 3.98E-12 |
| LRRK2 | -2.20 | 9.67E-08 |
| LSP1 | -3.43 | 2.04E-15 |
| LST1 | -2.74 | 3.78E-11 |
| LTB | -4.01 | 6.09E-18 |
| LTBP1 | 2.26 | 5.23E-14 |
| LY96 | -3.13 | 6.42E-14 |
| LYL1 | -2.21 | 2.44E-11 |
| LYN | -3.59 | 3.72E-16 |
| MAFB | -2.48 | 1.74E-10 |
| MAGED4B | 2.13 | 1.13E-11 |
| MAP2K3 | -2.08 | 2.82E-09 |
| MAP3K8 | -2.05 | 3.53E-10 |
| MBP | -3.41 | 1.04E-14 |
| MCAM | 2.00 | 2.75E-10 |
| MDFI | 2.18 | 5.15E-17 |
| MEI1 | -2.53 | 7.56E-10 |
| MFNG | -2.75 | 1.43E-14 |
| MGAM | -2.35 | 3.62E-12 |
| MILR1 | -2.11 | 3.76E-15 |
| MME | -2.39 | 3.12E-14 |
| MMP25 | -3.07 | 3.85E-09 |
| MMP9 | -2.84 | 1.49E-12 |
| MNDA | -3.35 | 1.17E-11 |
| MPEG1 | -3.22 | 2.60E-12 |
| MPZL3 | -2.58 | 4.51E-13 |
| MRC1 | -2.05 | 8.89E-09 |
| MRVI1 | -3.00 | 6.39E-12 |
| MS4A4A | -2.49 | 5.36E-13 |
| MS4A6A | -2.93 | 4.80E-15 |
| MS4A7 | -2.27 | 9.38E-09 |
| MT1DP | 2.67 | 1.80E-19 |
| MX2 | -2.71 | 1.53E-10 |
| MXD1 | -2.60 | 6.61E-12 |
| MYLIP | -2.22 | 4.95E-12 |
| MYO1F | -2.36 | 4.56E-10 |
| NAMPT | -3.14 | 7.74E-15 |
| NCF1 | -3.22 | 4.68E-11 |
| NCF2 | -3.44 | 2.21E-12 |
| NCF4 | -3.07 | 7.62E-15 |
| NCKAP1L | -2.25 | 5.04E-13 |
| NFAM1 | -3.45 | 8.26E-18 |
| NFE2 | -3.55 | 1.09E-12 |
| NFKBIZ | -2.32 | 1.25E-12 |
| NHSL2 | -2.19 | 3.79E-10 |
| NKG7 | -3.07 | 7.73E-16 |
| NKX1-2 | -2.84 | 1.99E-16 |
| NLRC3 | -2.30 | 1.46E-08 |
| NLRC4 | -2.80 | 7.28E-13 |
| NLRP12 | -3.01 | 4.03E-10 |
| NLRP3 | -2.75 | 1.51E-13 |
| NOD2 | -2.36 | 4.41E-11 |
| NOS2 | 2.65 | 1.01E-18 |
| NPC1L1 | -2.01 | 4.24E-08 |
| NPL | -2.37 | 4.45E-11 |
| NRGN | -2.27 | 4.73E-08 |
| NTS | -2.23 | 6.95E-11 |
| NUAK2 | -2.10 | 4.64E-15 |
| NUDT10 | 2.36 | 1.06E-27 |
| OASL | -2.06 | 3.83E-11 |
| OLFML2B | -2.19 | 1.66E-13 |
| OSM | -2.86 | 1.09E-13 |
| OTOA | -2.22 | 4.33E-09 |
| P2RY13 | -2.31 | 5.05E-12 |
| P2RY8 | -2.03 | 1.10E-07 |
| PAGE2B | -2.45 | 3.88E-21 |
| PARVG | -2.40 | 1.55E-10 |
| PCK1 | 3.23 | 9.16E-26 |
| PCYT1B | 2.54 | 2.13E-16 |
| PDE4B | -2.33 | 3.96E-10 |
| PDE5A | 2.08 | 2.69E-17 |
| PDLIM3 | 2.51 | 4.77E-15 |
| PDZD2 | 2.23 | 1.26E-17 |
| PDZK1IP1 | -2.73 | 7.54E-10 |
| PECAM1 | -3.17 | 6.31E-15 |
| PGA3 | -2.03 | 3.18E-16 |
| PGLYRP1 | -2.26 | 1.98E-11 |
| PI3 | -2.16 | 6.39E-07 |
| PIK3CD | -2.09 | 2.73E-10 |
| PIK3CG | -2.45 | 4.71E-11 |
| PLAC8 | -2.28 | 2.45E-13 |
| PLBD1 | -2.57 | 5.08E-08 |
| PLCB2 | -3.02 | 3.06E-12 |
| PLCG2 | -2.55 | 3.97E-12 |
| PLCXD3 | 2.33 | 3.17E-07 |
| PLEK | -3.40 | 5.30E-15 |
| PLEKHG3 | -2.39 | 1.19E-16 |
| PLOD2 | 2.51 | 4.79E-12 |
| PNCK | 3.36 | 4.84E-13 |
| POU2F2 | -2.24 | 2.30E-11 |
| PPBP | -2.76 | 4.51E-13 |
| PPP1R14C | 2.45 | 7.87E-22 |
| PRAM1 | -2.73 | 2.05E-10 |
| PRB1 | 2.24 | 2.66E-15 |
| PRB2 | 3.45 | 1.80E-19 |
| PRB3 | 2.05 | 1.24E-19 |
| PRB4 | 2.24 | 3.37E-13 |
| PRDM15 | 2.14 | 1.91E-13 |
| PREX1 | -3.50 | 8.96E-15 |
| PRKCB | -2.12 | 4.33E-09 |
| PROK2 | -4.30 | 5.90E-14 |
| PRR5 | -2.12 | 1.17E-10 |
| PSD3 | 2.05 | 2.92E-14 |
| PSTPIP1 | -2.59 | 8.72E-15 |
| PTAFR | -2.63 | 7.74E-15 |
| PTGER4 | -2.38 | 7.10E-10 |
| PTHLH | 2.18 | 4.66E-17 |
| PTPN6 | -2.22 | 8.06E-07 |
| PTPRC | -3.63 | 1.27E-18 |
| PTPRJ | -2.25 | 4.93E-07 |
| PYCARD | -2.11 | 4.69E-07 |
| Q3KR39 | -2.41 | 2.94E-11 |
| RAB11FIP1 | -2.72 | 5.68E-16 |
| RAB37 | -3.15 | 2.42E-21 |
| RAB6B | 2.09 | 6.36E-13 |
| RAC2 | -3.01 | 2.43E-16 |
| RASAL3 | -3.19 | 1.89E-17 |
| RASGRP2 | -2.67 | 2.50E-09 |
| RASSF5 | -3.24 | 6.18E-16 |
| RBP7 | -2.56 | 5.11E-23 |
| RCSD1 | -2.52 | 3.56E-14 |
| RGL4 | -3.42 | 1.55E-13 |
| RGS1 | -2.10 | 1.34E-10 |
| RGS18 | -3.19 | 8.23E-15 |
| RGS19 | -2.01 | 1.04E-11 |
| RHOBTB3 | 2.78 | 1.25E-22 |
| RIN3 | -2.10 | 2.08E-08 |
| RNASE6 | -2.37 | 1.77E-07 |
| RNASET2 | -2.19 | 3.29E-10 |
| RNF144B | -2.24 | 3.34E-17 |
| RSAD2 | -2.06 | 3.88E-10 |
| RTN4RL1 | 2.38 | 8.86E-18 |
| RUNX3 | -2.54 | 5.10E-13 |
| RYR2 | 2.86 | 4.77E-17 |
| S100A12 | -3.69 | 5.46E-16 |
| S100A4 | -3.33 | 2.16E-12 |
| S100P | -2.49 | 3.82E-14 |
| S1PR4 | -3.77 | 6.17E-17 |
| SAMSN1 | -2.09 | 9.35E-12 |
| SASH3 | -3.06 | 4.52E-15 |
| SCARNA9 | -2.25 | 4.50E-14 |
| SCG2 | 3.28 | 9.90E-18 |
| SCML4 | -2.25 | 1.38E-14 |
| SEC14L3 | -2.53 | 1.80E-19 |
| SECTM1 | -3.59 | 4.52E-18 |
| SELL | -2.13 | 9.14E-13 |
| SELPLG | -2.26 | 1.74E-15 |
| SEMA3A | 2.08 | 4.29E-14 |
| SEMA4D | -2.57 | 1.10E-14 |
| SEMA5B | 2.34 | 2.26E-12 |
| SERPINA3 | 2.27 | 2.26E-18 |
| SERPINA5 | 2.46 | 3.85E-20 |
| SERPING1 | -3.03 | 7.62E-14 |
| SH2D3C | -2.33 | 1.07E-12 |
| SIGLEC10 | -2.21 | 9.91E-15 |
| SIGLEC14 | -2.35 | 6.31E-12 |
| SIRPG | -2.75 | 2.44E-13 |
| SKAP1 | -2.40 | 8.88E-09 |
| SLA | -2.27 | 7.53E-15 |
| SLAMF7 | -2.40 | 1.12E-12 |
| SLC11A1 | -2.03 | 4.09E-07 |
| SLC15A1 | 2.91 | 5.34E-14 |
| SLC28A3 | 2.50 | 2.43E-13 |
| SLC2A1 | 2.10 | 1.57E-14 |
| SLC43A2 | -3.05 | 6.30E-15 |
| SLC4A1 | -2.72 | 5.55E-16 |
| SLC7A7 | -2.89 | 1.59E-13 |
| SLC8A1 | -2.52 | 1.41E-09 |
| SLCO2B1 | -2.38 | 5.76E-09 |
| SLED1 | -2.15 | 3.01E-08 |
| SLFN11 | -2.36 | 1.45E-10 |
| SLPI | -2.22 | 3.45E-08 |
| SMAD6 | 2.19 | 1.74E-19 |
| SMOC2 | 2.74 | 1.01E-20 |
| SNCA | -2.07 | 3.79E-10 |
| SNX10 | -2.02 | 4.03E-15 |
| SORL1 | -3.48 | 3.03E-15 |
| SPI1 | -3.23 | 1.26E-18 |
| SPON2 | 2.43 | 1.65E-12 |
| SQRDL | -2.79 | 6.61E-12 |
| SRMS | -2.10 | 1.57E-11 |
| SSH2 | -2.04 | 8.64E-13 |
| ST6GAL1 | -2.77 | 2.57E-13 |
| ST6GAL2 | 3.25 | 3.69E-19 |
| ST8SIA4 | -2.42 | 1.44E-09 |
| STAB1 | -2.24 | 3.53E-15 |
| STEAP4 | -2.22 | 2.77E-11 |
| SUSD4 | 2.01 | 3.75E-08 |
| SYK | -3.07 | 5.67E-17 |
| SYNDIG1 | 3.76 | 1.69E-16 |
| TAC3 | 2.80 | 2.22E-15 |
| TAGAP | -3.81 | 1.77E-12 |
| TBC1D10C | -3.40 | 1.06E-13 |
| TCL1A | -2.84 | 1.44E-10 |
| TESC | -2.77 | 9.90E-14 |
| TFAP2A | -2.44 | 2.47E-12 |
| TGFBI | -2.60 | 7.40E-14 |
| TGFBR2 | -2.52 | 2.07E-09 |
| THBD | -3.10 | 1.11E-11 |
| TLR1 | -3.36 | 5.60E-15 |
| TLR2 | -2.79 | 1.67E-10 |
| TLR4 | -2.87 | 9.34E-13 |
| TLR8 | -2.69 | 2.51E-09 |
| TMC8 | -2.44 | 4.44E-06 |
| TMEM114 | 3.01 | 4.09E-19 |
| TMEM132C | 3.09 | 6.21E-17 |
| TMEM154 | -2.78 | 1.29E-13 |
| TMEM71 | -2.91 | 1.07E-13 |
| TNC | 3.17 | 5.40E-15 |
| TNF | -2.44 | 9.78E-10 |
| TNFAIP2 | -2.77 | 1.98E-14 |
| TNFAIP6 | -2.31 | 5.56E-10 |
| TNFAIP8L2 | -2.87 | 8.26E-11 |
| TNFRSF8 | -2.01 | 1.05E-09 |
| TNFSF10 | -3.44 | 1.54E-16 |
| TNFSF13 | -2.05 | 1.78E-08 |
| TNFSF13B | -2.91 | 5.81E-11 |
| TNFSF14 | -3.05 | 3.55E-16 |
| TNRC18 | -2.81 | 1.05E-11 |
| TRAF3IP3 | -2.06 | 2.27E-10 |
| TREM1 | -2.37 | 1.65E-10 |
| TRIM58 | -3.36 | 1.09E-16 |
| TTBK1 | 2.27 | 8.80E-14 |
| TTTY16 | -4.04 | 1.06E-14 |
| ULBP1 | 3.99 | 3.87E-24 |
| VAMP8 | -2.36 | 3.07E-13 |
| VAV1 | -3.05 | 5.64E-16 |
| VAV3 | -2.21 | 4.29E-14 |
| VNN1 | -2.15 | 2.08E-09 |
| VNN2 | -2.98 | 3.96E-13 |
| VSTM1 | -2.12 | 1.77E-10 |
| VWA1 | -3.45 | 2.04E-15 |
| VWC2 | 2.26 | 1.48E-17 |
| WDFY4 | -3.06 | 1.64E-14 |
| WIPF1 | -2.74 | 5.40E-16 |
| WISP1 | 2.24 | 1.11E-17 |
| WNT3A | 2.28 | 1.76E-13 |
| XLOC_005327 | -2.29 | 2.60E-11 |
| XLOC_008144 | -2.28 | 1.03E-10 |
| XLOC_009375 | -2.67 | 1.42E-12 |
| XLOC_l2_007928 | 2.73 | 7.96E-18 |
| YPEL3 | -2.23 | 7.71E-09 |
| ZNF235 | -2.10 | 3.11E-10 |
