# Supplementary material for: Transcriptomic Analysis and Meta-Analysis of Human Granulosa and Cumulus Cells
Source: PLoS One. 2015 Aug 27;10(8):e0136473. doi: 10.1371/journal.pone.0136473 (PMC4552299; doi:10.1371/journal.pone.0136473)
Supplement: S2 Table — (DOCX) [file pone.0136473.s004.docx]

**S2 Table: Top 500 differentially expressed genes with higher expression in GC.**

| **Gene symbol** | **RP/Rsum** | **pfp** |
| --- | --- | --- |
| FGG | 5429 | 152.701 |
| NTS | 9774 | 114.181 |
| DUSP6 | 7767 | 50.868 |
| AOX1 | 7779 | 43.766 |
| IRF8 | 5058 | 2.apr |
| RGS18 | 12148 | 41.234 |
| EVI2B | 15960 | 39.904 |
| COL15A1 | 75 | 39.378 |
| TRIM22 | 12524 | 38.857 |
| ANKRD22 | 6438 | 37.829 |
| VCAM1 | 13705 | 36.188 |
| LYN | 4263 | 35.866 |
| TSPAN8 | 7438 | 35.416 |
| ANPEP | 472 | 35.241 |
| CTSC | 3894 | 35.015 |
| MMP10 | 12076 | 34.695 |
| FOS | 11602 | 34.452 |
| HCAR3 | 10347 | 34.316 |
| SHC4 | 10771 | 34.171 |
| ARHGDIB | 11367 | 34.053 |
| LY96 | 1414 | 33.798 |
| ENPP2 | 11954 | 33.167 |
| IL1B | 3299 | 32.873 |
| NFKBIZ | 2386 | 32.801 |
| CD97 | 3067 | 32.709 |
| HDC | 3058 | 32.543 |
| CSTA | 1063 | 32.514 |
| PLEK | 10702 | 32.382 |
| MARC2 | 10092 | 32.032 |
| GMFG | 1521 | 31.934 |
| LAPTM5 | 7472 | 31.797 |
| DOCK8 | 7898 | 31.618 |
| CYBB | 13852 | 31.188 |
| CASP1 | 10009 | 30.968 |
| HLA-DPA1 | 10696 | 30.862 |
| A2M | 2541 | 29.846 |
| GCA | 7961 | 29.819 |
| EGR2 | 5020 | 29.547 |
| LCP1 | 712 | 29.442 |
| PROK2 | 13917 | 29.033 |
| LYVE1 | 10738 | 28.944 |
| ARHGAP9 | 16246 | 28.917 |
| CARD16 | 7336 | 28.874 |
| GIMAP2 | 9980 | 28.659 |
| HCLS1 | 6675 | 28.592 |
| C5AR1 | 9487 | 28.577 |
| RARRES2 | 13750 | 28.567 |
| FCGR2A | 4507 | 28.404 |
| MAP3K8 | 2458 | 28.137 |
| CD53 | 11755 | 28.093 |
| SLC7A7 | 5304 | 27.981 |
| NCF2 | 6023 | 27.823 |
| AQP9 | 720 | 27.798 |
| FPR2 | 13524 | 27.709 |
| TGFBI | 8046 | 27.284 |
| FOSB | 8728 | 27.254 |
| UCP2 | 8121 | 27.052 |
| LITAF | 7417 | 27.042 |
| PPBP | 81 | 27.008 |
| SQRDL | 5368 | 26.941 |
| PGAP2 | 10019 | 26.908 |
| CCR1 | 3465 | 26.768 |
| MOCOS | 2735 | 26.611 |
| KLF2 | 3153 | 26.474 |
| INPP5D | 8369 | 26.339 |
| PSMB9 | 13495 | 26.238 |
| CD93 | 11400 | 26.147 |
| TAGAP | 3666 | 26.134 |
| S100A12 | 171 | 26.108 |
| CD163 | 12956 | 26.078 |
| ASAH1 | 8112 | 26.043 |
| GIMAP4 | 3375 | 26.038 |
| OSBPL6 | 2820 | 25.966 |
| FBP1 | 10556 | 25.723 |
| DHRS9 | 11144 | 25.716 |
| CYTIP | 9692 | 25.644 |
| TNFSF10 | 1731 | 25.636 |
| HBQ1 | 9712 | 25.541 |
| SORL1 | 6648 | 25.526 |
| PLBD1 | 15260 | 25.507 |
| HIST2H2BE | 9345 | 25.445 |
| HCK | 10700 | 25.246 |
| IFIT1 | 13562 | 25.206 |
| LTB | 7081 | 25.196 |
| LRP4 | 6686 | 25.053 |
| MPEG1 | 3086 | 25.035 |
| ACSS3 | 6510 | 25.014 |
| CCL5 | 9115 | 24.999 |
| KCNMA1 | 238 | 24.973 |
| IMPA2 | 2794 | 24.869 |
| IL18 | 842 | 24.842 |
| TLR1 | 1059 | 24.817 |
| CSN1S1 | 1626 | 24.778 |
| NR4A2 | 9766 | 24.657 |
| AOAH | 11192 | 24.648 |
| THBD | 6679 | 24.643 |
| PREX1 | 14035 | 24.566 |
| C16orf54 | 14909 | 24.549 |
| CX3CR1 | 2163 | 24.481 |
| FGL2 | 7706 | 24.428 |
| RBP7 | 7348 | 24.413 |
| FAM49A | 6362 | 24.412 |
| KLRB1 | 2192 | 24.398 |
| MMP9 | 2991 | 24.354 |
| FLI1 | 4173 | 24.316 |
| FREM2 | 11279 | 24.274 |
| GZMA | 1486 | 24.266 |
| MYLIP | 14854 | 24.252 |
| MRC1 | 15935 | 24.235 |
| APBB1IP | 8751 | 24.233 |
| VAMP8 | 8990 | 24.187 |
| KRT1 | 1666 | 24.137 |
| IL10RA | 3273 | 24.006 |
| ARHGAP25 | 6618 | 23.983 |
| PSMB8 | 5230 | 23.908 |
| CD14 | 13101 | 23.902 |
| GPR183 | 518 | 23.865 |
| TNFRSF21 | 10135 | 23.783 |
| IFIT2 | 14015 | 23.759 |
| CCR2 | 286 | 23.735 |
| CCL2 | 147 | 23.634 |
| SECTM1 | 12242 | 23.564 |
| FYB | 10431 | 23.536 |
| CD52 | 1996 | 23.519 |
| GABRP | 10381 | 23.518 |
| ACSL1 | 2077 | 23.461 |
| PTPRC | 1407 | 23.366 |
| SNX10 | 8387 | 23.305 |
| S1PR4 | 6396 | 23.303 |
| BTG2 | 12829 | 23.197 |
| IGSF6 | 8688 | 23.173 |
| HLA-DRA | 128 | 23.125 |
| LCP2 | 11990 | 23.095 |
| GIMAP7 | 6943 | 23.085 |
| C10orf54 | 9563 | 23.085 |
| STAT4 | 9265 | 23.082 |
| ITGAL | 4832 | 23.076 |
| CHST15 | 8413 | 23.059 |
| TGFBR2 | 14332 | 23.036 |
| CREG1 | 14919 | 23.005 |
| GZMB | 2177 | 22.982 |
| PPM1L | 15314 | 22.981 |
| LMO2 | 4055 | 22.978 |
| ALAS2 | 11677 | 22.968 |
| GMPR | 6284 | 22.918 |
| HBM | 10118 | 22.884 |
| CXCR2 | 8918 | 22.857 |
| SYK | 1427 | 22.851 |
| NFE2 | 3542 | 22.841 |
| APOBR | 15881 | 22.839 |
| GM2A | 9442 | 22.837 |
| GLT1D1 | 12980 | 22.821 |
| TREM1 | 7429 | 22.803 |
| PTGS2 | 3887 | 22.803 |
| ITGAM | 6312 | 22.721 |
| HLA-DMA | 7841 | 22.687 |
| EPN3 | 7707 | 22.677 |
| KLF4 | 11700 | 22.671 |
| PYCARD | 9054 | 22.559 |
| AMDHD1 | 14666 | 22.554 |
| FPR3 | 12529 | 22.547 |
| CCR7 | 14421 | 22.543 |
| CMTM2 | 10375 | 22.486 |
| ITLN1 | 410 | 22.477 |
| C1orf162 | 15845 | 22.459 |
| HLA-DMB | 9693 | 22.403 |
| RNASE6 | 12527 | 22.355 |
| NFAM1 | 14076 | 22.352 |
| ANXA1 | 1031 | 22.294 |
| TBC1D10C | 6223 | 22.287 |
| GIMAP6 | 16091 | 22.277 |
| IER2 | 13206 | 22.228 |
| FGD3 | 6259 | 22.195 |
| PDK4 | 1134 | 22.157 |
| SLC16A7 | 2063 | 22.137 |
| TLR4 | 12736 | 22.106 |
| S100A4 | 5378 | 22.091 |
| GIMAP1 | 2648 | 22.083 |
| PLCB2 | 3231 | 21.976 |
| CYB5A | 141 | 21.972 |
| LRRC25 | 2806 | 21.917 |
| NLRP3 | 8931 | 21.867 |
| NCF4 | 1058 | 21.838 |
| MEST | 5288 | 21.836 |
| SELENBP1 | 935 | 21.821 |
| SPI1 | 11810 | 21.753 |
| VNN2 | 4506 | 21.706 |
| RASAL3 | 9665 | 21.694 |
| IL2RB | 9729 | 21.662 |
| CLEC1B | 8586 | 21.609 |
| RGL4 | 15419 | 21.608 |
| VAV1 | 6906 | 21.595 |
| ANKRD33 | 15027 | 21.539 |
| HIST1H1C | 7931 | 21.525 |
| MANSC1 | 15536 | 21.445 |
| TNFRSF11B | 1624 | 21.443 |
| BST1 | 10155 | 21.419 |
| EMR3 | 15176 | 21.409 |
| ICAM3 | 5717 | 21.408 |
| ENPP3 | 6342 | 21.393 |
| MSR1 | 6155 | 21.371 |
| RGS1 | 8502 | 21.334 |
| KCNE3 | 6885 | 21.306 |
| PLTP | 8987 | 21.279 |
| TMEM71 | 6364 | 21.259 |
| C10orf11 | 3904 | 21.245 |
| GLUL | 3539 | 21.209 |
| HBEGF | 7739 | 21.178 |
| SOCS3 | 7932 | 21.158 |
| CEBPA | 4163 | 21.141 |
| GBP5 | 5769 | 21.116 |
| AKR1B1 | 479 | 21.115 |
| NKG7 | 15588 | 21.109 |
| CAMP | 3720 | 21.087 |
| CFD | 13088 | 21.071 |
| NLRP12 | 1435 | 21.022 |
| BIN2 | 11545 | 21.022 |
| PPL | 6775 | 20.966 |
| SLC43A2 | 15868 | 20.965 |
| RASSF5 | 8920 | 20.952 |
| SLFN11 | 11494 | 20.903 |
| CTSH | 4981 | 20.899 |
| SASH3 | 11694 | 20.887 |
| LRMP | 5874 | 20.886 |
| MRVI1 | 3698 | 20.876 |
| ALPL | 14169 | 20.876 |
| LST1 | 13305 | 20.875 |
| NCKAP1L | 13581 | 20.853 |
| PDE4B | 8505 | 20.836 |
| FCAR | 8767 | 20.831 |
| MS4A6A | 778 | 20.825 |
| SELL | 4997 | 20.755 |
| USP53 | 8214 | 20.746 |
| CITED1 | 382 | 20.728 |
| PIK3C2B | 2829 | 20.659 |
| ADAM8 | 2504 | 20.604 |
| CXCL2 | 8817 | 20.592 |
| OSR2 | 7979 | 20.577 |
| TEK | 6315 | 20.572 |
| CD36 | 2253 | 20.565 |
| HEMGN | 12971 | 20.533 |
| TESC | 9323 | 20.527 |
| LIPH | 4741 | 20.525 |
| ABHD5 | 14879 | 20.522 |
| TCL1A | 1922 | 20.499 |
| LGMN | 11338 | 20.498 |
| FGR | 4353 | 20.488 |
| RAB11FIP1 | 11022 | 20.465 |
| ADAP2 | 15139 | 20.454 |
| TPCN1 | 12195 | 20.432 |
| FAM117A | 9051 | 20.415 |
| TLR8 | 3668 | 20.412 |
| S100A8 | 14158 | 20.403 |
| CD300A | 3717 | 20.397 |
| ITK | 3693 | 20.386 |
| LPAR6 | 9151 | 20.377 |
| CST7 | 4513 | 20.345 |
| FCER1A | 4152 | 20.327 |
| LTA4H | 2716 | 20.315 |
| QPRT | 2990 | 20.283 |
| TNFAIP8L2 | 6064 | 20.282 |
| YPEL3 | 13653 | 20.258 |
| NAMPT | 1745 | 20.197 |
| PIK3CG | 10100 | 20.168 |
| EPHX2 | 12284 | 20.153 |
| GZMH | 3452 | 20.133 |
| RCSD1 | 5439 | 20.105 |
| CAT | 761 | 20.099 |
| GPR65 | 15552 | 20.054 |
| TMEM91 | 6976 | 20.043 |
| VAV3 | 3920 | 20.018 |
| BCL2A1 | 2861 | 19.995 |
| ITGB2 | 1112 | 19.989 |
| AIF1 | 11088 | 19.985 |
| SAMSN1 | 15582 | 19.952 |
| LPCAT3 | 4371 | 19.919 |
| AHSP | 8348 | 19.899 |
| INSL3 | 10804 | 19.892 |
| CCDC85A | 2452 | 19.881 |
| CXCR1 | 14422 | 19.809 |
| SLC9A3R1 | 8212 | 19.794 |
| CORO1A | 8966 | 19.792 |
| LILRA5 | 15733 | 19.774 |
| NRGN | 4932 | 19.767 |
| HSD17B1 | 3662 | 19.767 |
| JUNB | 2440 | 19.754 |
| TMEM154 | 6025 | 19.713 |
| FLJ30901 | 5343 | 19.711 |
| ABCA6 | 1469 | 19.693 |
| CLEC10A | 13131 | 19.687 |
| FFAR2 | 6839 | 19.672 |
| LGALS2 | 1795 | 19.658 |
| ARHGAP15 | 7156 | 19.619 |
| STAT1 | 2833 | 19.606 |
| OGFRL1 | 3740 | 19.602 |
| VNN1 | 14269 | 19.591 |
| IL18RAP | 3952 | 19.589 |
| EVI2A | 13393 | 19.585 |
| PHEX | 1189 | 19.582 |
| OLFML2B | 9914 | 19.517 |
| F13A1 | 945 | 19.512 |
| CLDN1 | 16073 | 19.447 |
| LRG1 | 13580 | 19.437 |
| GNS | 12763 | 19.436 |
| HCST | 6634 | 19.434 |
| EPB41L4B | 6729 | 19.422 |
| GNPDA1 | 3742 | 19.414 |
| CD86 | 8855 | 19.411 |
| DOCK11 | 14889 | 19.394 |
| CD33 | 12328 | 19.377 |
| TNF | 12955 | 19.358 |
| DENND2D | 8526 | 19.352 |
| PYGL | 4749 | 19.345 |
| TRPC3 | 11669 | 19.317 |
| MFNG | 4090 | 19.309 |
| BASP1 | 11371 | 19.307 |
| EGR1 | 2497 | 19.294 |
| MLKL | 14334 | 19.209 |
| OMA1 | 601 | 19.146 |
| DUSP1 | 3403 | 19.137 |
| CRYL1 | 5730 | 19.115 |
| DPEP2 | 14772 | 19.089 |
| SLC40A1 | 5213 | 19.017 |
| STAB1 | 2217 | 19.013 |
| C19orf59 | 6495 | 18.996 |
| P2RY13 | 4950 | 18.977 |
| KRT23 | 3303 | 18.939 |
| GIMAP8 | 4543 | 18.935 |
| LALBA | 1671 | 18.933 |
| CYTH4 | 14629 | 18.933 |
| CCNL1 | 2766 | 18.931 |
| SLCO2B1 | 11451 | 18.925 |
| SLC9A9 | 10235 | 18.912 |
| LY86 | 13502 | 18.888 |
| PLCG2 | 405 | 18.867 |
| CLEC7A | 14087 | 18.866 |
| CD300LF | 3965 | 18.858 |
| ITGAX | 2653 | 18.828 |
| PDZK1IP1 | 10395 | 18.827 |
| ANXA3 | 922 | 18.797 |
| FCN1 | 3956 | 18.785 |
| ARSE | 7864 | 18.771 |
| CECR1 | 14979 | 18.756 |
| EMB | 8315 | 18.746 |
| RNF144B | 344 | 18.726 |
| STX11 | 1893 | 18.717 |
| SH2D3C | 2742 | 18.707 |
| CD27 | 8079 | 18.678 |
| ARHGAP30 | 14086 | 18.666 |
| ABTB1 | 513 | 18.648 |
| CD74 | 8696 | 18.614 |
| SCPEP1 | 11120 | 18.569 |
| FAM65B | 12002 | 18.567 |
| DAPP1 | 4063 | 18.557 |
| P2RY8 | 2156 | 18.538 |
| NOD2 | 10648 | 18.423 |
| TMEM173 | 15533 | 18.421 |
| PLA2G7 | 2233 | 18.413 |
| CCBE1 | 11574 | 18.397 |
| FGFBP2 | 4425 | 18.379 |
| GAMT | 5598 | 18.376 |
| FCGR2B | 8092 | 18.365 |
| CPPED1 | 3635 | 18.348 |
| RNASET2 | 1822 | 18.318 |
| SLC25A37 | 9233 | 18.316 |
| LRRK2 | 11254 | 18.312 |
| HLA-F | 8701 | 18.306 |
| CHSY3 | 9897 | 18.305 |
| C1QA | 12497 | 18.293 |
| PTPN6 | 2783 | 18.278 |
| ARRB2 | 13104 | 18.249 |
| MYO1F | 6583 | 18.241 |
| VSIG4 | 3540 | 18.241 |
| CTBS | 10909 | 18.219 |
| STEAP4 | 5045 | 18.216 |
| AHR | 1899 | 18.208 |
| SSH2 | 3646 | 18.203 |
| SKAP1 | 307 | 18.173 |
| CD3G | 13139 | 18.147 |
| BTK | 5489 | 18.125 |
| GRB14 | 2064 | 18.125 |
| C15orf39 | 15363 | 18.113 |
| STRADB | 4209 | 18.053 |
| IL1RN | 390 | 18.036 |
| LILRA3 | 7819 | 18.021 |
| GNG2 | 3120 | 18.016 |
| GPR84 | 7036 | 17.998 |
| PSTPIP1 | 8103 | 17.952 |
| CSF3R | 8753 | 17.909 |
| IL2RG | 5194 | 17.908 |
| CSK | 5390 | 17.887 |
| MEI1 | 5049 | 17.856 |
| S100A9 | 7183 | 17.823 |
| FPR1 | 1577 | 17.816 |
| HVCN1 | 2797 | 17.809 |
| DGAT2 | 11515 | 17.807 |
| SPP1 | 527 | 17.792 |
| MCCC2 | 15468 | 17.778 |
| CMTM7 | 5624 | 17.775 |
| TNFSF13B | 8082 | 17.771 |
| CLEC4A | 8991 | 17.749 |
| PLEKHG3 | 1228 | 17.739 |
| SLAMF7 | 235 | 17.735 |
| TNFAIP2 | 5418 | 17.722 |
| OTUD1 | 12809 | 17.711 |
| OASL | 489 | 17.691 |
| PRAM1 | 9312 | 17.664 |
| SLA | 8672 | 17.663 |
| CSF2RA | 3350 | 17.625 |
| ELMO1 | 2578 | 17.543 |
| LCK | 2537 | 17.542 |
| PTGER4 | 8504 | 17.538 |
| KCNJ15 | 259 | 17.504 |
| KIAA0226L | 14127 | 17.498 |
| ACP5 | 2157 | 17.494 |
| CXorf21 | 14821 | 17.441 |
| AK7 | 2876 | 17.439 |
| CR1 | 1645 | 17.431 |
| LYL1 | 10757 | 17.412 |
| SLPI | 9249 | 17.411 |
| DEPTOR | 7930 | 17.406 |
| RUNX3 | 2869 | 17.392 |
| HLA-DPB1 | 208 | 17.385 |
| APOC1 | 7387 | 17.348 |
| SYTL3 | 8969 | 17.296 |
| RAB37 | 14089 | 17.211 |
| SIGLEC10 | 14926 | 17.208 |
| PGLYRP1 | 7194 | 17.176 |
| CDC42EP4 | 8599 | 17.165 |
| C1QC | 7186 | 17.137 |
| ICAM1 | 3009 | 17.136 |
| LSP1 | 8691 | 17.127 |
| SLC16A6 | 16065 | 17.115 |
| PTPLAD2 | 12401 | 17.074 |
| NPY | 10531 | 17.063 |
| LDHD | 14282 | 17.038 |
| IL12RB2 | 12550 | 17.035 |
| NR4A1 | 7528 | 17.005 |
| MX2 | 134 | 17.002 |
| SPRR3 | 5301 | 16.969 |
| CXCL14 | 12561 | 16.965 |
| CD5 | 13642 | 16.963 |
| SERPINE1 | 5480 | 16.956 |
| OSM | 3406 | 16.908 |
| HMHA1 | 9409 | 16.905 |
| C1QB | 10400 | 16.874 |
| ITPRIP | 1828 | 16.853 |
| APOBEC3B | 9497 | 16.761 |
| VSTM1 | 15621 | 16.731 |
| USP33 | 13751 | 16.718 |
| FERMT3 | 2963 | 16.666 |
| HLA-DPB2 | 4171 | 16.589 |
| RPA4 | 4233 | 16.511 |
| RXRA | 14896 | 16.469 |
| DUSP2 | 545 | 16.326 |
| GPR182 | 2044 | 15.632 |
| MXD1 | 7440 | 15.538 |
| MNDA | 8712 | 13.523 |
| IL8 | 6704 | 13.371 |
| EGR3 | 1930 | 12.195 |
| BCO2 | 9245 | 2.814 |
| QPCT | 13001 | 2.468 |
| TLR2 | 3432 | 2.455 |
| RARRES3 | 4112 | 2.447 |
| ZFP36 | 9145 | 2.388 |
| CCL4 | 8025 | 2.327 |
| CD48 | 8459 | 2.326 |
| CTSS | 3770 | 2.192 |
| KCNJ2 | 11963 | 2.178 |
| MS4A4A | 11378 | 2.176 |
| GK5 | 10768 | 2.158 |
| PLD2 | 7683 | 2.152 |
| TNFAIP6 | 14784 | 2.068 |
| TNFSF14 | 2800 | 2.043 |
| SP110 | 542 | 2.042 |
| EPB42 | 9660 | 2.037 |
| CA1 | 10452 | 2.014 |
| IRF1 | 8388 | 2.014 |
| DECR1 | 4782 | 1.976 |
| ERP27 | 9978 | 1.938 |
| CCR5 | 5739 | 1.925 |
| ICAM2 | 7351 | 1.869 |
| ENTPD3 | 10693 | 1.848 |
| APOA1 | 2845 | 1.839 |
| LILRB1 | 60 | 1.825 |
| MAFB | 4634 | 1.815 |
| LILRB2 | 3279 | 1.797 |
| CD69 | 4609 | 1.795 |
| PI3 | 13067 | 1.794 |
| LILRA2 | 8141 | 1.768 |
| EMR2 | 13910 | 1.764 |
| LEF1 | 637 | 1.758 |
| HK3 | 3816 | 1.747 |
| MME | 8084 | 1.747 |
| GZMK | 12945 | 1.737 |
| PIM1 | 9927 | 1.722 |
| PTAFR | 4308 | 1.719 |
| S100P | 8585 | 1.709 |
| CITED4 | 10258 | 1.621 |
| TRIB1 | 15736 | 1.619 |
