# Supplementary material for: Transcriptomic Analysis and Meta-Analysis of Human Granulosa and Cumulus Cells
Source: PLoS One. 2015 Aug 27;10(8):e0136473. doi: 10.1371/journal.pone.0136473 (PMC4552299; doi:10.1371/journal.pone.0136473)
Supplement: S3 Table — (DOCX) [file pone.0136473.s005.docx]

**S3 Table: Top 500 differentially expressed genes with higher expression in CC.**

| **Gene symbol** | **RP/Rsum** | **pfp** |
| --- | --- | --- |
| KCNH2 | 4036 | 0.5318 |
| FGF11 | 6605 | 0.4403 |
| ELL2 | 7649 | 0.649 |
| TMEM176A | 15190 | 0.5416 |
| TRPC1 | 8061 | 0.6493 |
| MVD | 12602 | 0.675 |
| MT1E | 174 | 0.7172 |
| IL1R1 | 2490 | 0.6118 |
| RAI14 | 15324 | 0.6053 |
| ZNF836 | 13039 | 0.6615 |
| MEX3B | 12485 | 0.6715 |
| ZNF234 | 6293 | 0.6248 |
| RPS6KA6 | 6856 | 0.5736 |
| TCEAL2 | 15561 | 0.5626 |
| APBA1 | 843 | 0.6087 |
| MTMR2 | 959 | 0.5592 |
| CPNE7 | 2292 | 0.6172 |
| PPP4R4 | 11826 | 0.6461 |
| NPFF | 7591 | 0.6029 |
| SNX30 | 11399 | 0.5906 |
| USP11 | 7918 | 0.658 |
| DIAPH3 | 2346 | 0.6316 |
| FZD3 | 8754 | 0.592 |
| ZDHHC8P1 | 16117 | 0.616 |
| KIAA1522 | 7831 | 0.5847 |
| MYL9 | 15751 | 0.6268 |
| NPY5R | 626 | 0.649 |
| ZNF555 | 6044 | 0.6332 |
| HS3ST3A1 | 6687 | 0.583 |
| HILPDA | 14075 | 0.8122 |
| ZBTB38 | 8739 | 0.6072 |
| NPC1 | 655 | 0.5614 |
| SLC7A1 | 13423 | 0.5991 |
| TTYH1 | 15655 | 0.6292 |
| POPDC2 | 12817 | 0.6111 |
| CADM1 | 2045 | 0.6076 |
| CRY1 | 14484 | 0.5436 |
| CORO1C | 12259 | 0.5312 |
| TPM2 | 8698 | 0.6311 |
| MAML2 | 13931 | 0.543 |
| GJA4 | 13717 | 0.6955 |
| AFF3 | 4453 | 0.54 |
| CDK2AP1 | 8546 | 0.6081 |
| PLSCR4 | 15766 | 0.6886 |
| ARL10 | 11831 | 0.6257 |
| SORT1 | 3811 | 0.5556 |
| SLIT2 | 10237 | 0.5605 |
| CLCN6 | 7490 | 0.5669 |
| REPS1 | 15825 | 0.5939 |
| CRYAB | 4466 | 0.6185 |
| C12orf68 | 11458 | 0.5363 |
| SOCS1 | 12047 | 0.6748 |
| KDM6A | 7879 | 0.5742 |
| MXRA5 | 4148 | 0.4826 |
| ALOX5 | 4151 | 0.4275 |
| RIMS4 | 15484 | 0.6632 |
| GPC1 | 11429 | 0.5607 |
| SLC46A2 | 12483 | 0.5761 |
| PPFIA4 | 13969 | 0.5632 |
| KCND2 | 2570 | 0.6213 |
| CNN1 | 6985 | 0.6287 |
| ANGPT2 | 5457 | 0.6338 |
| LMO1 | 12400 | 0.6687 |
| PTGFR | 734 | 0.6098 |
| ADAM12 | 3719 | 0.535 |
| SMAD7 | 16010 | 0.5283 |
| RHPN2 | 11084 | 0.5777 |
| NPY1R | 10853 | 0.6902 |
| TMEM130 | 8405 | 0.6818 |
| GDF11 | 14167 | 0.6184 |
| PPP1R3C | 9418 | 0.746 |
| CACNA1H | 4357 | 0.706 |
| PDGFA | 1178 | 0.5529 |
| KCNJ8 | 8729 | 0.6334 |
| TCF21 | 13628 | 0.6272 |
| RNF144A | 5994 | 0.6106 |
| DCTN4 | 3353 | 0.6067 |
| PDLIM7 | 8556 | 0.5809 |
| TNNT1 | 12276 | 0.4757 |
| SH3D21 | 504 | 0.6229 |
| FHOD3 | 14451 | 0.5799 |
| ARMCX2 | 11420 | 0.627 |
| GNG4 | 12599 | 0.5944 |
| PRDM15 | 10935 | 0.6 |
| DUOX1 | 3256 | 0.6177 |
| CACNA2D1 | 4394 | 0.5896 |
| TUBB2B | 3595 | 0.4767 |
| CHRDL2 | 299 | 0.59 |
| DNER | 5684 | 0.6509 |
| TOX2 | 7761 | 0.4987 |
| BNIP3 | 12106 | 0.5967 |
| ERC1 | 685 | 0.6004 |
| LAYN | 10607 | 0.626 |
| FAM57A | 14637 | 0.573 |
| LIMD1 | 1456 | 0.5756 |
| SORD | 5959 | 0.6172 |
| METTL7B | 10969 | 0.5599 |
| FAM110B | 567 | 0.5715 |
| TSHZ2 | 10613 | 0.5393 |
| CLIC6 | 13809 | 0.6037 |
| PRKCI | 4185 | 0.5809 |
| ENDOD1 | 11976 | 0.6082 |
| PRICKLE2 | 3096 | 0.7222 |
| DAG1 | 10811 | 0.6026 |
| MT1F | 2046 | 0.5995 |
| HOXC9 | 6959 | 0.5933 |
| MAP1LC3A | 3025 | 0.537 |
| TES | 4808 | 0.5603 |
| HIVEP3 | 5668 | 0.5559 |
| PCNT | 6079 | 0.5629 |
| PSME4 | 108 | 0.505 |
| ZMAT4 | 5709 | 0.5637 |
| ZSWIM5 | 1462 | 0.5801 |
| RRS1 | 15901 | 0.642 |
| TNFRSF10D | 10415 | 0.6041 |
| ARHGAP10 | 10907 | 0.5343 |
| CRADD | 2302 | 0.5651 |
| DCBLD2 | 7263 | 0.5889 |
| SEMA3B | 14191 | 0.5588 |
| CHL1 | 9402 | 0.527 |
| NEDD4 | 2693 | 0.4963 |
| SPATS2 | 6700 | 0.5515 |
| CAMK2B | 12297 | 0.6301 |
| SYT11 | 6205 | 0.5615 |
| HERC5 | 312 | 0.5081 |
| ZNF275 | 14207 | 0.5679 |
| PPP1R14A | 15355 | 0.4427 |
| GMNN | 13661 | 0.5547 |
| WIPI1 | 4071 | 0.5461 |
| COL4A4 | 4065 | 0.6484 |
| IL11 | 9182 | 0.6538 |
| SIGLEC11 | 14266 | 0.5364 |
| NCS1 | 10749 | 0.6004 |
| FAM162A | 12346 | 0.5864 |
| HAPLN1 | 2641 | 0.6063 |
| B4GALT5 | 1363 | 0.4809 |
| SNTG2 | 9339 | 0.61 |
| ANKRD18A | 2268 | 0.6453 |
| CXCL6 | 6705 | 0.4408 |
| SLC35F2 | 4946 | 0.5915 |
| CHST1 | 1245 | 0.5942 |
| HTRA3 | 6760 | 0.5793 |
| RNF182 | 12996 | 0.4907 |
| PDPN | 6263 | 0.6282 |
| SEMA6A | 15156 | 0.545 |
| MYRIP | 14959 | 0.5936 |
| DUOXA1 | 14038 | 0.5957 |
| PEG10 | 14440 | 0.5225 |
| PDK3 | 8482 | 0.5273 |
| TUBB3 | 5300 | 0.6071 |
| SHCBP1 | 6184 | 0.5941 |
| FRMPD1 | 9034 | 0.6566 |
| SLC6A8 | 8638 | 0.5422 |
| GPC4 | 4986 | 0.5494 |
| EBF4 | 16023 | 0.5758 |
| ASAP2 | 1614 | 0.4824 |
| DAAM1 | 8154 | 0.492 |
| LRRC38 | 302 | 0.6492 |
| GPRASP1 | 11058 | 0.5789 |
| ABCC3 | 3099 | 0.5209 |
| DCBLD1 | 2546 | 0.5446 |
| PLXDC2 | 14555 | 0.5205 |
| HIST1H1A | 11128 | 0.6068 |
| CNTN3 | 10311 | 0.4974 |
| UAP1L1 | 15699 | 0.5362 |
| TMEM229B | 15861 | 0.5107 |
| TMEM163 | 10950 | 0.5284 |
| MTSS1 | 10157 | 0.5497 |
| MLLT11 | 15224 | 0.5472 |
| COL6A1 | 2106 | 0.5949 |
| TNFSF4 | 11911 | 0.5856 |
| ITM2A | 15842 | 0.6219 |
| VASN | 13143 | 0.592 |
| ABTB2 | 11638 | 0.577 |
| ADRA2A | 14541 | 0.5777 |
| RORB | 3094 | 0.4496 |
| ADAMTS16 | 3665 | 0.5564 |
| AR | 576 | 0.5463 |
| GPC6 | 5263 | 0.5137 |
| AGPAT6 | 2441 | 0.5918 |
| PHKA2 | 11048 | 0.5436 |
| ZBTB7C | 6997 | 0.5634 |
| MYO1D | 6125 | 0.5643 |
| NT5DC3 | 969 | 0.5475 |
| ULBP3 | 2812 | 0.5583 |
| FZD7 | 8658 | 0.5357 |
| SERPINH1 | 2712 | 0.5446 |
| LGR4 | 8392 | 0.5768 |
| MT2A | 15791 | 0.614 |
| PDLIM4 | 9369 | 0.5458 |
| ARHGAP20 | 10999 | 0.6258 |
| FBXL20 | 13132 | 0.5174 |
| HOXD10 | 11036 | 0.5854 |
| ITGB3 | 9911 | 0.5781 |
| GLDN | 15120 | 0.5404 |
| RCN3 | 14211 | 0.5673 |
| SCN9A | 6459 | 0.4019 |
| SEMA7A | 2235 | 0.6036 |
| DACT1 | 14658 | 0.5217 |
| ADRB1 | 11102 | 0.6474 |
| GPM6B | 13069 | 0.549 |
| PARP11 | 12521 | 0.5225 |
| LUM | 5773 | 0.5582 |
| STC2 | 6882 | 0.4956 |
| RDX | 1914 | 0.4874 |
| CLIP3 | 6747 | 0.5638 |
| MAGI3 | 16064 | 0.5733 |
| PITX2 | 5367 | 0.5978 |
| SH3RF1 | 12883 | 0.5409 |
| NFATC4 | 8703 | 0.5694 |
| WIPF3 | 12337 | 0.4785 |
| GRIK3 | 9276 | 0.5165 |
| INSIG1 | 557 | 0.5189 |
| PAMR1 | 9500 | 0.5517 |
| PPP1R12B | 606 | 0.4658 |
| CLDN3 | 2556 | 0.5897 |
| DUOXA2 | 13611 | 0.529 |
| BICD1 | 10032 | 0.5119 |
| DPY19L4 | 1988 | 0.5343 |
| TDRD10 | 9146 | 0.5003 |
| ACAT2 | 6300 | 0.5824 |
| PRKCA | 6325 | 0.5549 |
| SQLE | 282 | 0.5494 |
| GEM | 14318 | 0.3951 |
| LOXL2 | 1009 | 0.5798 |
| SLC7A11 | 3621 | 0.4593 |
| HMGCS1 | 10407 | 0.5716 |
| NUDT10 | 7929 | 0.598 |
| C12orf23 | 7468 | 0.532 |
| HHIP | 10331 | 0.5479 |
| LDLR | 762 | 0.5874 |
| INHBA | 676 | 0.4654 |
| KCNG1 | 5161 | 0.5228 |
| SLCO2A1 | 1634 | 0.4442 |
| NPTX1 | 15169 | 0.5736 |
| AQP2 | 6790 | 0.4824 |
| PCDH19 | 9535 | 0.5143 |
| FSTL4 | 11807 | 0.5609 |
| ITGA2 | 12584 | 0.5274 |
| PTGIS | 6444 | 0.5198 |
| SNTB1 | 5474 | 0.5266 |
| EPB41L4A | 8651 | 0.533 |
| LGALS1 | 8713 | 0.4462 |
| CDH12 | 12101 | 0.4343 |
| ATP2B4 | 4168 | 0.4916 |
| THBS1 | 8905 | 0.4135 |
| NACAD | 4596 | 0.4745 |
| SYN1 | 4611 | 0.5203 |
| RNF145 | 11583 | 0.5065 |
| TYMS | 3026 | 0.5715 |
| GPT2 | 6892 | 0.5205 |
| ABLIM3 | 12315 | 0.447 |
| MAP1A | 6951 | 0.5254 |
| SOCS2 | 7450 | 0.5399 |
| GSTM1 | 6275 | 0.6042 |
| EFCAB4B | 12448 | 0.5251 |
| NAP1L5 | 11548 | 0.5147 |
| CD24 | 10928 | 0.5122 |
| SFRP4 | 9575 | 0.3822 |
| CRH | 1253 | 0.5204 |
| RRAD | 3805 | 0.4355 |
| HPS3 | 3481 | 0.5213 |
| MAGED2 | 4559 | 0.5425 |
| MAPK4 | 8827 | 0.6202 |
| LAPTM4B | 10688 | 0.5492 |
| BEND5 | 15149 | 0.5603 |
| C18orf54 | 7044 | 0.5094 |
| KLHL24 | 11576 | 0.4967 |
| COL1A1 | 2994 | 0.5152 |
| LEPREL1 | 13776 | 0.4899 |
| PTPRH | 14822 | 0.4448 |
| PGR | 665 | 0.543 |
| IGDCC4 | 6060 | 0.5428 |
| IHH | 9845 | 0.6011 |
| C7 | 4147 | 0.4646 |
| CRTAC1 | 13892 | 0.5489 |
| AKR1C1 | 2250 | 0.4988 |
| COL21A1 | 11163 | 0.4523 |
| ACTA2 | 5185 | 0.5243 |
| BDNF | 4770 | 0.481 |
| DLG5 | 7200 | 0.5285 |
| DDIT4 | 1003 | 0.5191 |
| STXBP5L | 10457 | 0.4973 |
| COL6A3 | 541 | 0.4895 |
| GCG | 7346 | 0.4667 |
| P4HA2 | 2928 | 0.5153 |
| GSTA3 | 10503 | 0.4456 |
| HMGCR | 4699 | 0.459 |
| DCLK1 | 2140 | 0.465 |
| CEACAM21 | 1743 | 0.5155 |
| UBASH3B | 11562 | 0.4749 |
| VSTM2L | 5660 | 0.4839 |
| KLHDC8A | 13237 | 0.4684 |
| SMAD6 | 14411 | 0.5583 |
| LRIG3 | 14213 | 0.5514 |
| NNMT | 7014 | 0.4491 |
| MDFI | 14315 | 0.5874 |
| KLK1 | 11100 | 0.5205 |
| NPTX2 | 14575 | 0.4375 |
| HPCAL4 | 10338 | 0.4184 |
| ZNF469 | 15746 | 0.4699 |
| PRB3 | 84 | 0.5202 |
| HSPA12A | 16157 | 0.5175 |
| HAS2 | 635 | 0.4492 |
| SPOCD1 | 10756 | 0.3894 |
| LINGO2 | 10130 | 0.3955 |
| MUM1L1 | 13670 | 0.4619 |
| SLC25A18 | 12174 | 0.5059 |
| CDH11 | 3663 | 0.4532 |
| TANC2 | 12549 | 0.4422 |
| PEMT | 2407 | 0.3865 |
| WISP2 | 10051 | 0.4778 |
| PDLIM3 | 9164 | 0.502 |
| PTCHD1 | 6425 | 0.4418 |
| NT5DC2 | 11311 | 0.5198 |
| DUOX2 | 3706 | 0.3724 |
| SEMA5B | 10577 | 0.5236 |
| TGFB1I1 | 6963 | 0.3796 |
| TCEAL7 | 15664 | 0.3953 |
| CBLB | 7655 | 0.3925 |
| IGFBP7 | 8233 | 0.5067 |
| KIF5C | 11643 | 0.4478 |
| MMP16 | 2688 | 0.4157 |
| FAM110C | 12393 | 0.4301 |
| TRO | 5579 | 0.4728 |
| CLU | 5770 | 0.4761 |
| GLIPR1 | 9519 | 0.3744 |
| COLQ | 11631 | 0.4755 |
| RNF43 | 13182 | 0.4932 |
| EPHB1 | 9186 | 0.3457 |
| KLF12 | 13541 | 0.4148 |
| PDZD2 | 253 | 0.4315 |
| RAB33A | 3616 | 0.4261 |
| HTR7 | 5634 | 0.4703 |
| GREM1 | 10886 | 0.4005 |
| F2RL1 | 14316 | 0.451 |
| C2CD4B | 14709 | 0.563 |
| IL7R | 7166 | 0.3079 |
| NLGN4X | 10812 | 0.4441 |
| SLC15A1 | 4918 | 0.455 |
| HMCN1 | 3357 | 0.4021 |
| MT3 | 2332 | 0.419 |
| ATF7IP2 | 12367 | 0.3918 |
| TMEM45A | 11555 | 0.4403 |
| PNMT | 11652 | 0.4058 |
| WISP1 | 1457 | 0.5036 |
| STAC | 6846 | 0.4043 |
| C3orf67 | 8922 | 0.4386 |
| FRMD5 | 6015 | 0.4146 |
| MT1M | 2542 | 0.3477 |
| MRC2 | 11769 | 0.4473 |
| PTHLH | 9647 | 0.4909 |
| SRPX | 1623 | 0.4252 |
| GFPT2 | 13990 | 0.3168 |
| TNNI3 | 9343 | 0.4013 |
| MAOB | 3606 | 0.4136 |
| DHH | 13823 | 0.4906 |
| NRIP3 | 2608 | 0.381 |
| EXT1 | 6269 | 0.349 |
| RGS5 | 73 | 0.4492 |
| NAP1L3 | 15405 | 0.4062 |
| PARM1 | 11727 | 0.4071 |
| DIRAS3 | 5488 | 0.3568 |
| ADAMTS14 | 7079 | 0.4403 |
| FBLN5 | 11096 | 0.3671 |
| CHGA | 10584 | 0.4829 |
| COL6A2 | 8328 | 0.4127 |
| NOS2 | 43 | 0.5087 |
| SOX9 | 1864 | 0.3669 |
| LXN | 10790 | 0.3432 |
| ACTA1 | 3007 | 0.4702 |
| RGS4 | 5992 | 0.2567 |
| FSCN1 | 8125 | 0.3747 |
| PLEKHH1 | 14597 | 0.3446 |
| AEBP1 | 6843 | 0.363 |
| PRB4 | 10326 | 0.383 |
| KRTAP13-1 | 15611 | 0.5044 |
| DCHS1 | 4797 | 0.3662 |
| CACNA1C | 6104 | 0.3577 |
| LIMS2 | 7740 | 0.3915 |
| AIM1 | 7636 | 0.3536 |
| DKFZp451A211 | 8510 | 0.4237 |
| RASD1 | 1078 | 0.4521 |
| GAL | 8869 | 0.3385 |
| ACOXL | 5426 | 0.3974 |
| EDIL3 | 10726 | 0.3134 |
| SEMA3A | 12122 | 0.3735 |
| CRHBP | 8326 | 0.4178 |
| ITGA1 | 4889 | 0.3802 |
| BMPER | 6082 | 0.339 |
| CD200 | 4488 | 0.353 |
| SHE | 14346 | 0.3675 |
| CCDC3 | 11900 | 0.4331 |
| CHGB | 2403 | 0.372 |
| F3 | 3864 | 0.4371 |
| DNM3 | 10096 | 0.3441 |
| FAM189A1 | 13869 | 0.3876 |
| RAB6B | 14433 | 0.3984 |
| PLA2G5 | 14243 | 0.3453 |
| PXDN | 3851 | 0.3334 |
| ARG2 | 874 | 0.363 |
| CYP19A1 | 3349 | 0.4179 |
| CBLN1 | 11384 | 0.3483 |
| TSPAN7 | 3222 | 0.3005 |
| FBXO32 | 5885 | 0.3066 |
| LIPG | 5898 | 0.2429 |
| GPR158 | 7502 | 0.3348 |
| NDP | 10 | 0.2493 |
| VCAN | 10037 | 0.3248 |
| BUB1 | 7652 | 0.3979 |
| CLMP | 4462 | 0.3642 |
| PDE5A | 5100 | 0.372 |
| DLX5 | 3365 | 0.3891 |
| CALB2 | 8808 | 0.2768 |
| TAC3 | 12625 | 0.38 |
| PCYT1B | 10575 | 0.3193 |
| MCAM | 5077 | 0.3155 |
| GJA5 | 3769 | 0.3082 |
| PPP1R14C | 3066 | 0.3048 |
| GPR56 | 2512 | 0.2867 |
| FAM150B | 6039 | 0.2989 |
| FABP3 | 454 | 0.3075 |
| ALPK2 | 11428 | 0.296 |
| CORO2A | 2514 | 0.3036 |
| ISM1 | 11404 | 0.3341 |
| GPRC5B | 7305 | 0.3121 |
| LTBP1 | 10005 | 0.2482 |
| CIB4 | 14200 | 0.3321 |
| FN1 | 5123 | 0.2972 |
| PLOD2 | 8900 | 0.2816 |
| SLC44A5 | 2899 | 0.5503 |
| CLSTN2 | 15946 | 0.2554 |
| GRIK2 | 9282 | 0.5878 |
| PCK1 | 5447 | 0.3335 |
| LOC283588 | 13605 | 0.5335 |
| GAP43 | 3765 | 0.2824 |
| SERPINA5 | 2486 | 0.2528 |
| ARSK | 2377 | 0.5874 |
| SLC28A3 | 11611 | 0.257 |
| KCNK3 | 12154 | 0.2497 |
| KIF26A | 14339 | 0.6068 |
| GABRA5 | 7298 | 0.5643 |
| SLC44A3 | 10521 | 0.5842 |
| LRAT | 5854 | 0.2897 |
| TRIO | 3683 | 0.5819 |
| ST6GAL2 | 7337 | 0.2357 |
| SMOC2 | 8782 | 0.2582 |
| PLCXD3 | 15972 | 0.2099 |
| PRICKLE1 | 1946 | 0.6034 |
| RHOBTB3 | 6981 | 0.2839 |
| FRRS1 | 13590 | 0.634 |
| FAM161A | 13884 | 0.5829 |
| DAPL1 | 12082 | 0.2721 |
| SPARCL1 | 5354 | 0.6456 |
| LOC100131541 | 3690 | 0.5215 |
| GRIN2A | 2495 | 0.4678 |
| CTSK | 6591 | 0.2492 |
| COL13A1 | 399 | 0.6062 |
| DISP1 | 14560 | 0.5756 |
| SUSD4 | 9964 | 0.4669 |
| PCDHB14 | 11741 | 0.547 |
| KIAA1210 | 8461 | 0.4369 |
| ACSS1 | 358 | 0.5497 |
| ARHGEF4 | 3074 | 0.5311 |
| ANG | 13061 | 0.4993 |
| GPX8 | 3433 | 0.5071 |
| GJA1 | 1631 | 0.4846 |
| FOXG1 | 1077 | 0.2379 |
| PAPPA | 14526 | 0.492 |
| MAGEL2 | 552 | 0.4826 |
| CILP | 11790 | 0.2048 |
| AMIGO2 | 12938 | 0.4337 |
| DOK5 | 14723 | 0.1718 |
| SLC1A3 | 13563 | 0.4714 |
| ARHGAP6 | 244 | 0.4851 |
| RYR2 | 11019 | 0.1491 |
| ENO2 | 3890 | 0.4072 |
| KIF1A | 10399 | 0.5247 |
| TMEM132C | 7631 | 0.1921 |
| SYNDIG1 | 15476 | 0.2214 |
| E2F7 | 6820 | 0.1737 |
| TIMP3 | 7169 | 0.4218 |
| FLRT3 | 14469 | 0.4348 |
| C3orf36 | 16170 | 0.3574 |
| PDGFC | 9028 | 0.4191 |
| FSTL3 | 6083 | 0.3316 |
| GABBR2 | 1110 | 0.4455 |
| LEFTY2 | 3727 | 0.4195 |
| PSD3 | 6397 | 0.3686 |
| GSTM3 | 1200 | 0.363 |
| EPYC | 16017 | 0.3022 |
| SERPINA3 | 5047 | 0.3335 |
| HTRA1 | 5222 | 0.1391 |
| TNC | 907 | 0.0916 |
| SCG2 | 9002 | 0.2333 |
| ULBP1 | 9723 | 0.2362 |
| PNCK | 9079 | 0.2137 |
| BEX1 | 12713 | 0.1688 |
| ACE2 | 20 | 0.1665 |
| IGFBP5 | 3189 | 0.1645 |
